# General Intelligence Framework to Predict Virus Adaptation Based on a Genome Language Model

**DOI:** 10.34133/research.0871

**Published:** 2025-09-30

**Authors:** Shu-Yang Jiang, Shi-Shun Zhao, Jun-Qing Wei, Sen Zhang, Zhongpeng Zhao, Yigang Tong, Wei Liu, Jianwei Wang, Tao Jiang, Jing Li

**Affiliations:** ^1^ College of Mathematics, Jilin University, Changchun, Jilin 130012, China.; ^2^ State Key Laboratory of Pathogen and Biosecurity, Academy of Military Medical Science, Beijing 100071, China.; ^3^ Beijing Advanced Innovation Center for Soft Matter Science and Engineering (BAIC-SM), College of Life Science and Technology, Beijing University of Chemical Technology, Beijing 100029, China.; ^4^ NHC Key Laboratory of Systems Biology of Pathogens and Christophe Merieux Laboratory, National Institute of Pathogen Biology, Chinese Academy of Medical Sciences and Peking Union Medical College, Beijing 100730, China.; ^5^ Key Laboratory of Respiratory Disease Pathogenomics, Chinese Academy of Medical Sciences and Peking Union Medical College, Beijing 100730, China.

## Abstract

Most human viral pandemics are caused by animal-originated viruses with human adaptation. It is challenging to infer adaptation from viral genes or their coded protein sequences, particularly when the data labels for modeling are inadequate or the input sequence to be predicted is incomplete. Here, we developed a semi-supervised General Intelligence framework to predict Virus Adaptation based on Language-model-embedded protein sequences (GIVAL) for blind input of virus sequences. The language model in GIVAL, named virus Bidirectional Encoder Representations from Transformers (vBERT), was pretrained for embedding using hidden Markov model-contextualized tokens of viral protein sequences. vBERT outperformed prevalent pretrained models like DNABERT-2, proteinBERT, ESM-2, Transformer, and Word2Vec on distinguishing viral proteins with various-grained labels, such as serotypes and single phenotype-altering mutation. The semi-supervised GIVAL obtained higher accuracy in virus adaptation prediction and better fault tolerance on raw labels in the training dataset, overcoming the obstacle of modeling with insufficient labels and predicting blind input. GIVAL was applicable to the adaptation prediction of diverse viruses. For influenza A viruses (IAVs), higher human adaptation was predicted for equine-origin H3N8 IAVs and bovine H5N1 IAVs with simulated mutations. For coronaviruses, GIVAL predicted an adaptation shift of receptor binding from Middle East respiratory syndrome–related coronavirus (MERS-CoV) receptor to severe acute respiratory syndrome coronavirus receptor of 2 recently reported MERS-CoV-like virus variants. For monkeypox viruses, GIVAL quantified an incremental adaptation shift of viral variants, matching the rise in human monkeypox cases. Summarily, GIVAL provides a generally intelligent framework for predicting virus adaptation based on its genotype, with the potential to extend to more genotype-to-phenotype prediction scenarios.

## Introduction

Most emerging or remerging virus epidemics are caused by zoonotic viruses [1], such as the last 5 influenza pandemics [2] caused by influenza A viruses (IAVs), the worldwide Coronavirus Disease 2019 (COVID-19) pandemic caused by severe acute respiratory syndrome coronavirus 2 (SARS-CoV-2) [3], and other global infectious disease outbreaks [4]. However, it is challenging to predict the cause of the next virus pandemic. It is well known that most mammal- or bird-prevalent viruses only occasionally cause spillover human infection and are incapable of causing sustained inter-human transmission or even launching a pandemic before being adapted to humans [5,6]. Such adaptation manifests the phenotypes of human-specific receptor binding, higher replication efficiency, antagonizing the host’s anti-virus immune response, more efficient transmission, and other phenotypes [6–9]. It is inspiring that viral phenotypes, including host adaptation, are determined by their genotypes and are thus theoretically predictable based on their genotypes [10–16]. However, it would be time-consuming for traditional strategies to explore the viral genotype–phenotype causality using reverse genetics and other molecular virological methods [11–13,17]. Given the urgency and importance of assessing the pandemic potential of mammalian or avian viruses, it is vital to build a method to predict the adaptation of a virus to human host based on its genotype.

Inspiring predictions of viral phenotypes, such as host adaptation, using deep learning or machine learning, have been reported based on viral genome information. Distinctive viral genomic compositional traits or encoded proteins accurately predicted the reservoir hosts and arthropod vectors [18], and the adaptation and transmission of IAVs [19,20], coronaviruses (CoVs) [21,22], and monkeypox virus [23]. Such genome distinctiveness has been biologically interpreted as immunomodulatory activity [6,24], viral virulence [25], and replication [26] and, thus, as host specific. Natural language processing (NLP) embedding of protein sequences examines the genotype–phenotype association in more detail. Language model-embedded proteins intelligently predicted signal peptides [27], phylogenetic relationships [28], subcellular localization [29], post-translation modification [30], and structural features [31]. Moreover, language-embedded viral proteins accurately predicted viral evolution and structural escape [32] and conservation and variant effects [33]. However, the insufficiency of adaptation or other phenotype labels for virus proteins was one of the main obstacles for supervised learning models [34]. Additionally, most current intelligent predictors were limited to specified viruses and specified virus genes within a narrow sequence length range [35,36]. Thus, more general intelligent predictors were anticipated.

The present study aimed to pretrain a general viral genome language embedder, named virus Bidirectional Encoder Representations from Transformers (vBERT), for general embedding of viral protein sequences, and then to build a generalized framework of General Intelligence to predict Virus Adaptation based on Language model (GIVAL), without specified training data, a specified sample label, and a specified trained model. Any input of viral protein sequence (full or segmented) will lead to an output list of mapped virus genotypes, automatically optimized labeled data for modeling, automatically in-time model training, and input-based virus adaptation prediction. It is worth mentioning that semi-supervised learning was conducted to obtain optimized labels based on data clustering, to train a supervised classifier, and to statistically infer the adaptation label based on the predicted classes and raw adaptation labels of the training data. Our framework provides general intelligence for genotype-to-phenotype prediction of viruses, such as viral adaptation. GIVAL and its language embedder were benchmarked and evaluated for their potential to generally predict viral adaptation based on their embedded proteins for CoVs, IAVs, and monkeypox viruses.

## Results

### Workflow of vBERT-based GIVAL to predict host adaptation of viruses

**Fig. 1.**
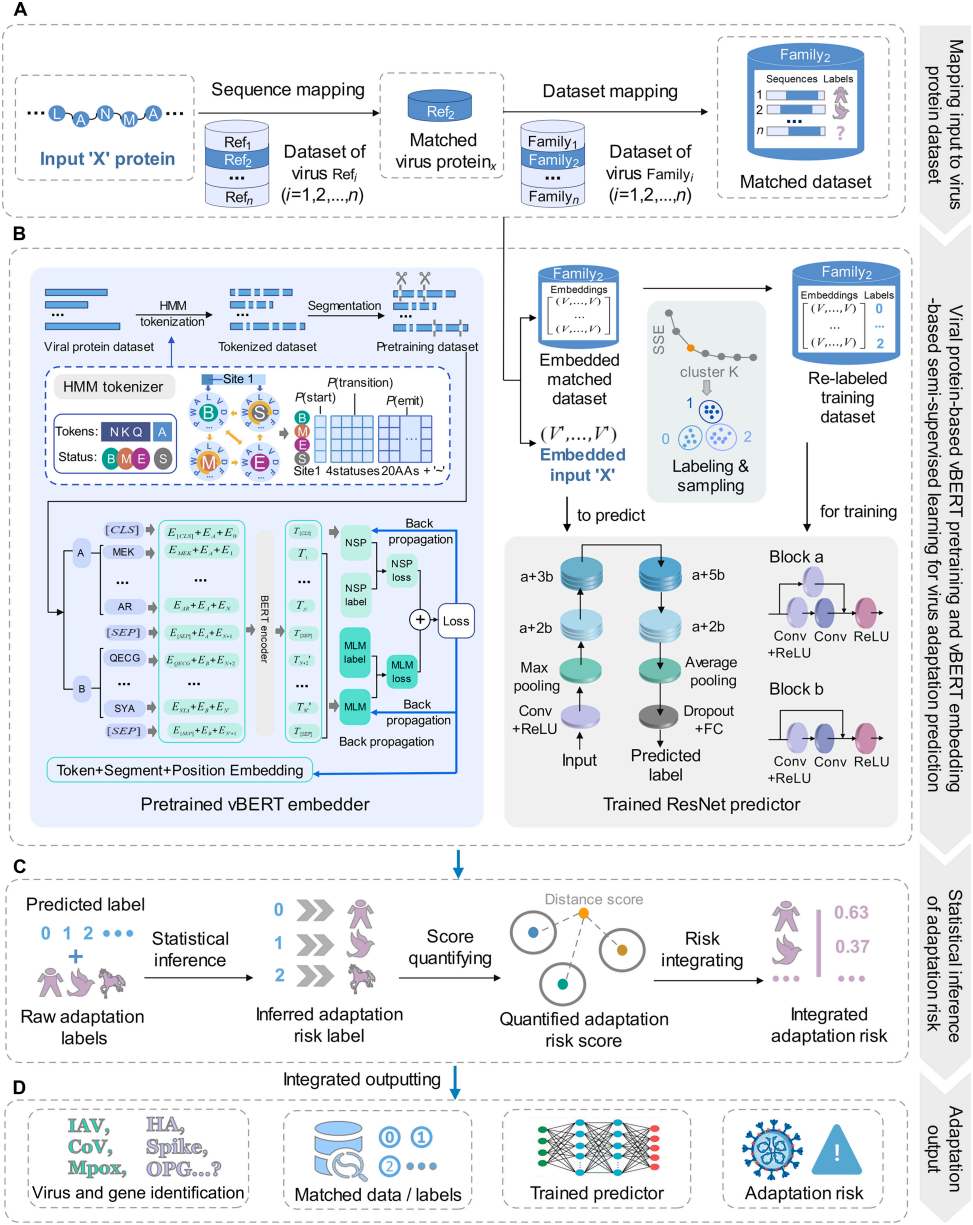
The pipeline of GIVAL to generally predict the adaptation risk of a virus based on vBERT embedding of its genotype. The pipeline of GIVAL can be divided into 4 parts. (A) Dataset intelligentization for input sequence identification and mapped dataset retrieval. (B) Label intelligentization and label-based sampling based on the vBERT embedding of the mapped dataset and model intelligentization by timely training a ResNet model with flexibly optimized labels for input sequence prediction. (C) Inference and quantifying of the adaptation risk of the input sequence based on the predicted label and raw annotations. (D) Integrated output of identified viral protein, flexible dataset with label, the input-customized model, and predicted results for the input sequence.

GIVAL intelligently predicted the input viral protein sequence or segment adaptation without a trained model based on specified data with labels. Firstly, after inputting the unknown viral sequence with a varied length, a customized BLAST+ mapping with the reference sequence dataset identified the viral protein and retrieved the mapped dataset (Fig. 1A). Secondly, the vBERT-embedded retrieved dataset was re-labeled and sampled to create the training dataset (Fig. 1B). Thirdly, a ResNet predictor was trained on the training dataset (Fig. 1B). Finally, the adaptation risk of the input sequence was statistically inferred and quantified based on the predicted label and raw annotations (Fig. 1C). The integrated results of the identified viral protein, input-customized datasets, labels, model, and predicted adaptation risk were output (Fig. 1D). GIVAL provided a semi-supervised framework for input sequence retrieval, dataset labeling and sampling, predictor training, and adaptation inference.

### Performance of hidden Markov model tokenization and vBERT embedding on virus proteins

**Fig. 2.**
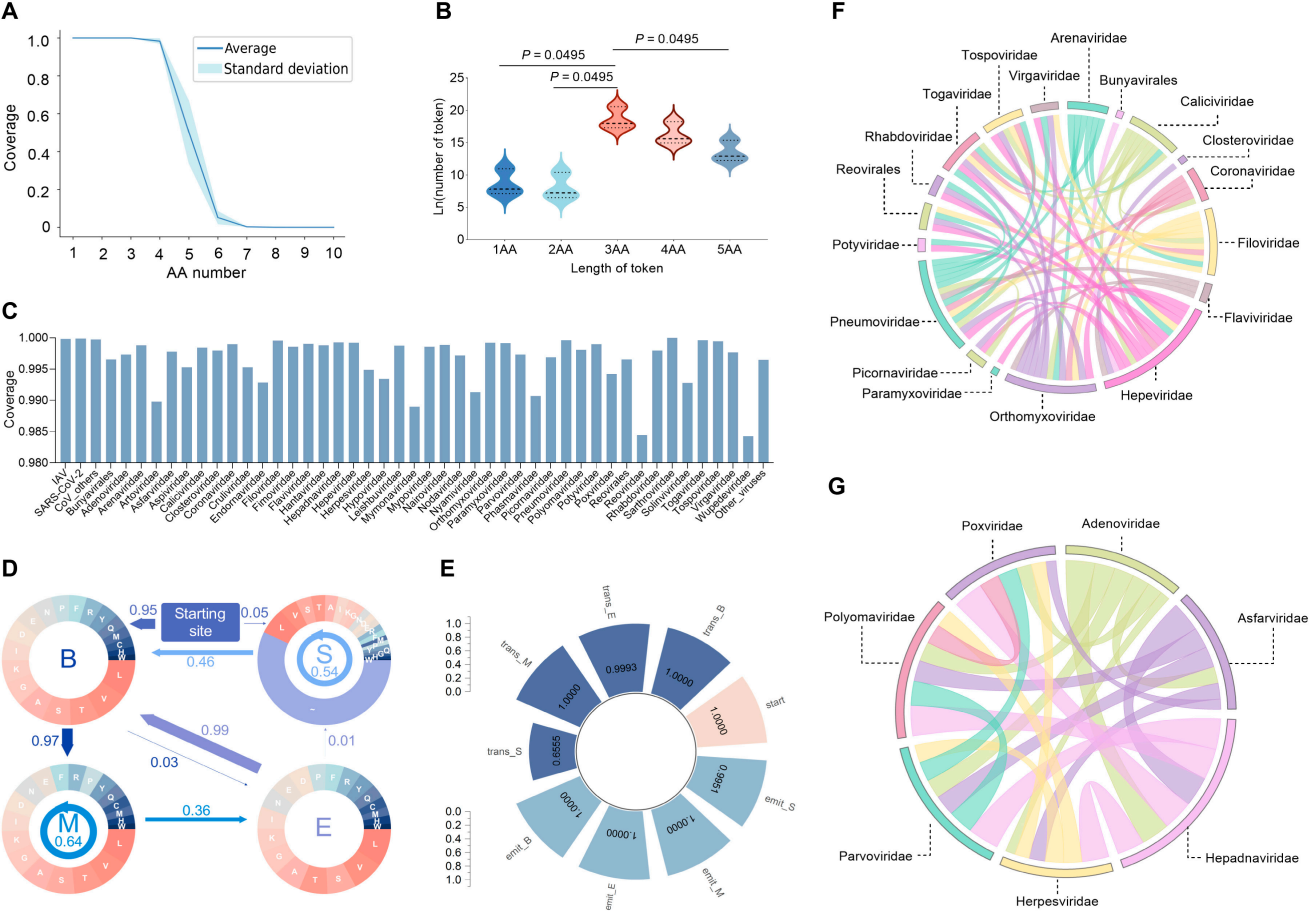
Performance of HMM tokenizer in vBERT on different datasets. (A) The coverage of 1- to 10-AA vocabularies in the sequences of the whole dataset, HMM tokenizer dataset, and vBERT pretraining dataset. (B) The natural logarithm of the number of tokens with 1 to 5 AAs in the HMM-tokenized vocabulary list of the whole dataset, HMM tokenizer dataset, and vBERT pretraining dataset. (C) The coverage with weight of the HMM vocabulary list for each type of viruses in the whole dataset. (D) The starting, emit, and transition probability of 20 types of AAs and other unknown characters for the HMM tokenizer. (E) The angle cosine between the parameter vectors of the HMM model based on the real and virtual dataset. Connection indexes of RNA viruses with value greater than 0.6 (F) and DNA viruses (G).

vBERT was pretrained for hidden Markov model (HMM)-tokenized viral protein sequences (Fig. S1A to E). The HMM tokenizer was trained based on the sampled dataset and optimized for the parameters. Firstly, viral family based and statistical sampling for the whole dataset (Figs. S2 and S3A and B) was validated and compared to random sampling (Fig. S3C). The more even number distribution of each viral family of vBERT pretraining dataset and HMM training dataset compared with the whole dataset were shown by the higher Simpson indexes (Fig. S3D). The absence of marked differences in the indexes within each family before and after sampling also reflected the rationality of sampling in preserving the genetic diversity while ensuring balanced representation of distinct sequence types (Fig. S3E). Secondly, the HMM tokenizer was evaluated. Statistical analysis indicated more than 99% coverage of 1- to 4-amino-acid (AA) tokens and a plunged coverage of 9% for 6-AA tokens (Fig. 2A). Therefore, the HMM tokenizer was trained using 1- to 5-AA tokens. The highest frequency of 3-AA tokens was followed by 4-AA and 5-AA tokens, with significantly fewer 1- or 2-AA tokens (*P* = 0.0495, Fig. 2B). The HMM-tokenized vocabulary list for vBERT pretraining indicated a weighted coverage of more than 98% and an average coverage of 92% for the whole dataset (Fig. 2C and Fig. S4A). Thirdly, probability parameters were optimized for the HMM tokenizer. A starting probability of {B: 0.95, M: 0, E: 0, S: 0.05}, a transition probability of {B: {M: 0.97, E: 0.03}, M: {M: 0.64, E: 0.36}, E: {B: 0.99, S: 0.01}, S: {S: 0.54, B: 0.46}}, and an emit probability of serially probability values were obtained (Fig. 2D and Table S1). Fourthly, the robustness of the HMM tokenizer was verified with the high consistency between the parameters based on the real and virtual datasets (Fig. 2E), the similarity of the frequency of the top 50 vocabularies among all major RNA and DNA (Fig. S4B and C) viral families, and the separation between the full-token frequency vectors for each RNA or DNA viral family (Fig. 2F and G).

**Fig. 3.**
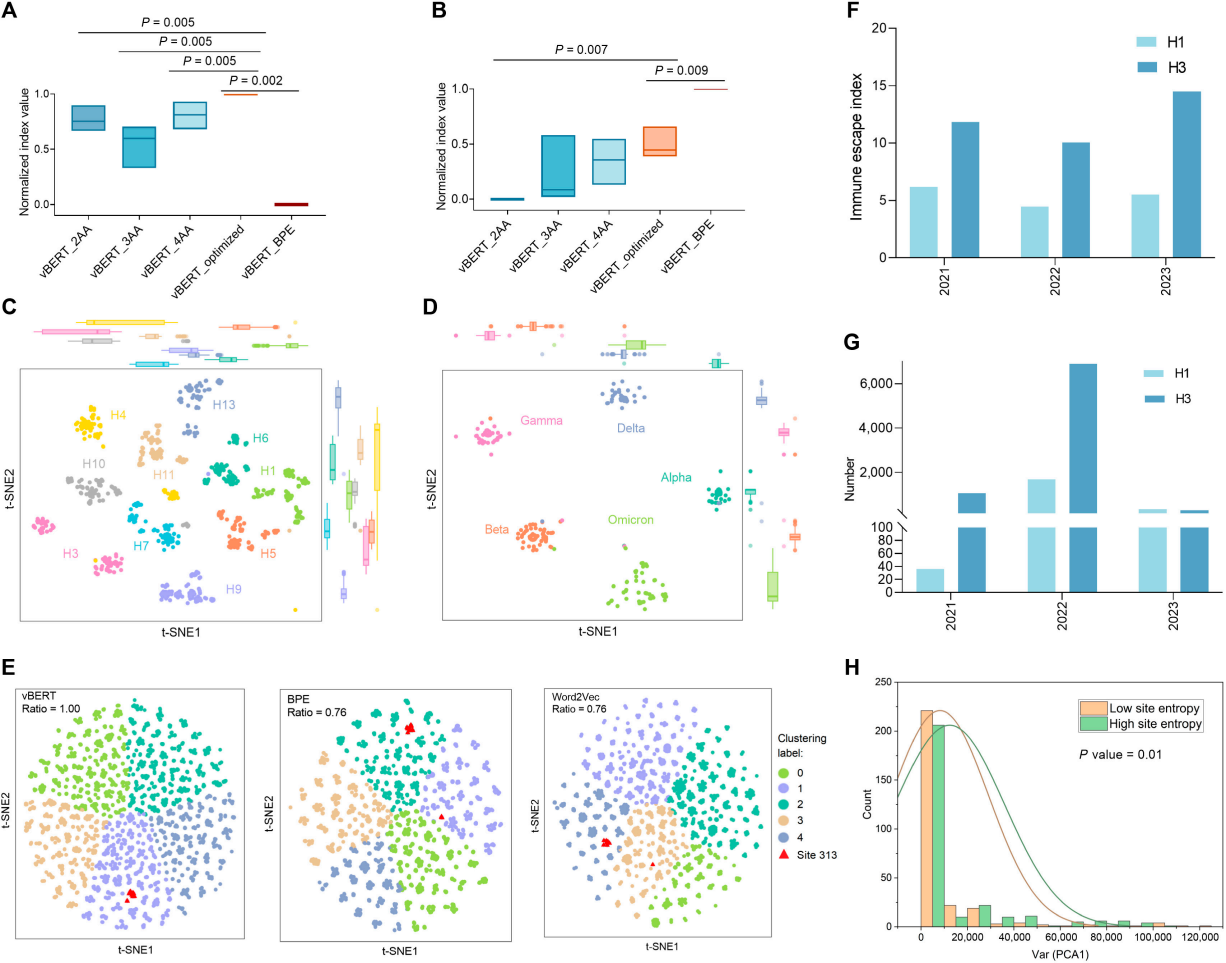
Embedding performance of vBERT and other pretrained models on various virus proteins. The embedding performance of the vBERT-optimized compared with models pretrained with 2 to 4 AAs as tokens and BPE tokenization, respectively, with normalized silhouette, CH, ARI, NMI, and one minus normalized DBI, on the SARS-CoV-2 Spike RBD (A) and IAV HA (B) test dataset. Reduced 2 components with t-SNE of vBERT-embedded IAV HA (C) and SARS-CoV-2 Spike RBD (D). Reduced 2 components with t-SNE of the vBERT-optimized-, vBERT-BPE-, and Word2Vec-embedded (E) tokens at site 313 and other tokens of IAV NP and the maximum ratio of the tokens at site 313 in the same cluster. The immune escape index calculated based on vBERT embedding (F) and the number of circulating H1 and H3 from 2021 to 2023 (G). (H) The PCA1 variance values of sites with high and low site entropy. The PCA1 variance values of sequences with each site singly mutated with 20 AAs were obtained based on the vBERT-embedded IAV HA DMS dataset.

vBERT was optimized for its parameters and benchmarked based on embedding performance. Firstly, the vBERT-optimized was pretrained based on the HMM-tokenized sequences, indicating better performance than the models pretrained with the fixed 2-AA, 3-AA, or 4-AA tokens in the ablation study of HMM tokenization. The vBERT-optimized performance was significantly higher for CoV receptor binding domain (RBD) (*P* < 0.05, Fig. 3A). A similar outperformance of the vBERT-optimized over the BERT with 2-AA tokens was also observed for IAV hemagglutinin (HA) (*P* < 0.05, Fig. 3B). The vBERT-optimized underperformed on the HA test dataset but outperformed vBERT-BPE (byte pair encoding) on the Spike RBD test dataset. However, vBERT-BPE exhibited marked performance instability across both datasets. Overall, among the 3 tokenization methods (2-4AA, BPE, and HMM), HMM demonstrated the most robust performance. Secondly, the vBERT-optimized was benchmarked at various granularities. The coarse-grained outperformance was verified with clearer intra-group clustering and inter-group separation (Fig. 3C and D), compared with Transformer, ESM-2, DNABERT-2, proteinBERT, and other models for parameter optimization (Figs. S5A to R and S6A to R, Table S2, and Table). Compared with the BERT models pretrained based on the whole and the simulated dataset, the outstanding performance of the vBERT-optimized confirmed that excessive homologous sequences were not conducive to embedding. Furthermore, the vBERT-optimized based on the BERT-base configuration performed better than the models based on smaller-scale configuration, such as BERT-tiny and BERT-medium. The fine-grained outperformance was demonstrated by the more balanced clustering and separation of vBERT-optimized-embedded tokens (Fig. 3E and Fig. S7A to D) covering biologically important AA sites of 16, 313, 319, or 357 in IAV nucleoprotein (NP) [37] than Word2Vec-embedded tokens (Fig. 3E and Fig. S7E to H) and vBERT-BPE-embedded tokens (Fig. 3E and Fig. S7I to L). The enriched semantics of domain–function association were shown by the clear separation between the vBERT embedding of the HMM tokens in hypervariable IAV HA1 domain and conserved HA2 domain, addressing that the HMM-based vBERT embedding was able to capture protein domain boundaries instead of merely focusing on token frequency (Fig. S8A to O). Thirdly, the embedding performance of the vBERT-optimized was further tested in a scenario to evaluate the immune escape of IAV and the singular AA variation of the DMS datasets. WHO-referred IAV H1 HA strains were closer to prevalent H1 strains (Fig. S9A), while the referred H3 strains were more dispersed from prevalent H3 strains (Fig. S9B), with the immune escape indexes (Fig. 3F) aligning with the number of reported H1 and H3 samples (Fig. 3G). The difference between high- and low-binding variants was observed for the principal component analysis (PCA) reduced value of the vBERT-optimized-embedded SARS-CoV-2 Spike, with singular AA variation at the site of 339, 449, 452, or 505 (*P* < 0.1, Fig. S9C). The 2 classes of IAVs with different preference entropies were also significantly different in PCA1 of the embedded HAs (*P* = 0.01, Fig. 3H). Taken together, the optimized vBERT performed well in multiple-grained scenarios, sensitive to discrimination of subtle semantics of virus proteins.

**Table.** Benchmarking of vBERT with other pretrained language models. The 5 clustering indexes (silhouette, CH, DBI, ARI, and NMI) of all models involved in benchmarking were calculated and compared. Average score represents the average of normalized silhouette, CH, ARI, and NMI values and one minus normalized DBI value. The optimal indexes were emphasized using boldface.

	Index	vBERT	Transformer	ESM-2	proteinBERT	DNABERT-2
IAV_HA	Silhouette	0.576	**0.590**	0.562	0.511	0.550
	CH	**3,348.260**	3,060.565	3,064.684	2,423.532	3,070.174
	DBI	**0.580**	0.636	0.683	0.721	0.636
	ARI	0.834	0.871	**0.906**	0.688	0.740
	NMI	0.905	0.933	**0.935**	0.812	0.823
Spike_RBD	Silhouette	0.870	0.779	0.581	0.707	**0.873**
	CH	**14,400.057**	4,262.676	1,165.237	1,019.655	10,420.517
	DBI	0.215	0.353	0.677	0.502	**0.192**
	ARI	**0.968**	0.961	0.771	0.784	0.963
	NMI	**0.955**	0.947	0.813	0.776	0.951
	Average score	**0.919**	0.762	0.382	0.086	0.678

### Generalization validation of GIVAL to predict virus adaptation

**Fig. 4.**
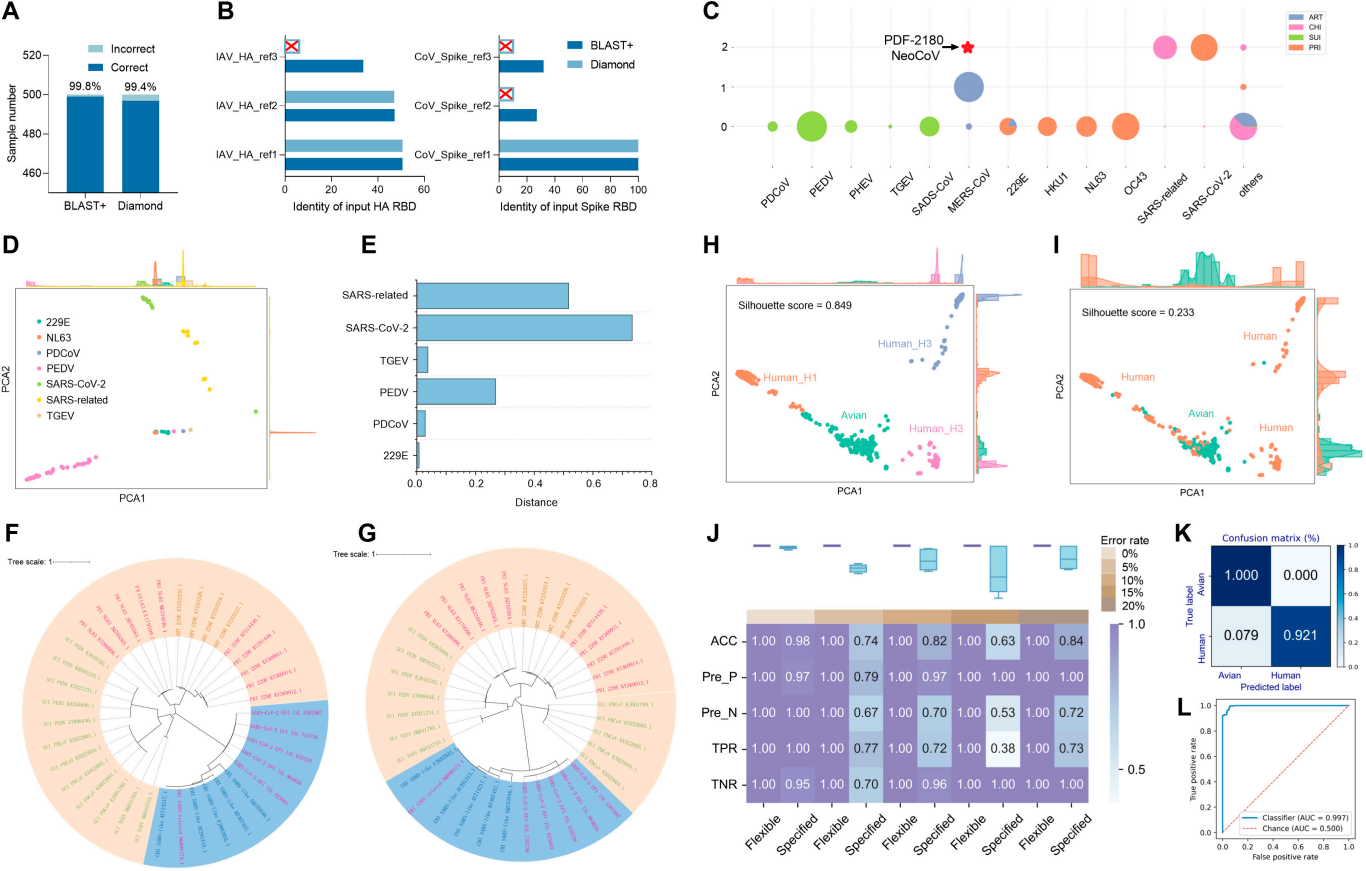
Performance of GIVAL on predicting adaptation risk of IAVs and CoVs. (A) The number of correctly and incorrectly mapped sequences in the test dataset based on customized BLAST+ and Diamond. The percentage represents the mapping accuracy. (B) The mapping identity between the input HA RBD and Spike RBD sequences and the mapped reference sequences (IAV_HA_ref1-3 are strain A/Jalna/NIV9436/2009, A/mallard/Sweden/101490/2009 and A/Louisiana/02/2017; CoV_Spike_ref1-3 are strain NC045512.2, KF530080.1 and MG596802.1). (C) The Artiodactyla (ART)-, Chiroptera (CHI)-, Suiformes (SUI)-, and Primates (PRI)-adaptive ratio of CoV Spike RBD sequences in each flexible label. The red star represents the predicted label of PDF-2180 and NeoCoV strain. Reduced 2 components with PCA of vBERT-embedded 229E, NL63, PDCoV, SARS-CoV-2, SARS-related, and TGEV Spike RBD sequences (D) and the distances between the PCA vectors of NL63 and others (E). Phylogenetic analysis on Spike RBD (F) and Spike complete sequences (G) of SUI, ART, CHI SARS-like, and PRI 229E, NL63, OC43, SARS-related, and SARS-CoV-2. Reduced 2 components with PCA of vBERT-embedded IAV HA training dataset based on flexible (H) or specified adaptive host (I) labels. (J) The performance of models based on flexible and specified labels with various error rates of training human H3 label. The confusion matrix (K) and ROC_AUC (L) of the model based on the input IAV HA RBD on the independent validation dataset.

The generalization performance of the input-specified GIVAL was validated using IAV HA, a segmented IAV HA RBD, and a segmented SARS-CoV-2 Spike RBD [38]. Firstly, BLAST+ mapping based on the reference sequences was verified with higher accuracy than Diamond on the test dataset and the 2 input sequences (Fig. 4A and B). Secondly, the outperformance of flexible labeling in GIVAL was shown in the ablation study compared with specified labeling. From the perspective of unsupervised learning, the flexible labels predicted by GIVAL based on RBD-covered Spike sequences generally divided CoV Spike sequences into 3 groups: Middle East respiratory syndrome coronaviruses (MERS-CoVs), SARS-CoV-2/SARS-related viruses, and others (Fig. 4C and Fig. S10A to C). Such prediction was consistent with the 3 types of receptor binding specificity of CoVs, dipeptidyl peptidase-4 (DPP4) for MERS-CoVs, angiotensin-converting enzyme 2 (ACE2) for SARS-CoV-2/SARS-related viruses, and aminopeptidase N (APN) for others [12,39,40], with one exception of NL63 in the third group and with ACE2 as a receptor, which was proven by the longer distance from NL63 to SARS-related than to TGEV, PEDV, PDCoV, and 229E (Fig. 4D and E) and phylogenetic analysis (Fig. 4F and G). The 2 strains of PDF-2180 and NeoCoV were predicted exactly to be ACE2-binding, rather than DPP4-binding (Fig. 4C and Fig. S10D), by our model, which was not distinguished by phylogenetic analysis (Fig. S10E). Our result was definitely consistent with the reported results [41]. Flexible labeling showed higher rationality with distinct separation of sequences (Fig. 4H) compared to the specified host labels (Fig. 4I). The flexible labels of HA RBD (Fig. S11A to D) generally divided the dataset into human-adaptive H1 and H3 and avian-adaptive samples. From the perspective of supervised learning, flexible labeling contributed to higher accuracy and raw label fault tolerance of the ResNet model than specified labeling (Fig. 4J and Fig. S12A to F), and the prediction of the model was verified with high accuracy in the confusion matrices and ROC_AUC of the 3 cross-validation sets of HA RBD, HA complete sequence, and Spike RBD (Figs. S13A to F, S14A to F, and S15A to F) and of the independent validation set of the HA RBD model (Fig. 4K and L). Tests on input with extremely short lengths exhibited that GIVAL showed high accuracy on segments with 40 and 50 AAs, while the prediction accuracy was relatively unstable at 30-AA segments (Fig. S16A to I). This might be caused by the overlapping local features of different types of sequences and the insufficient information-carrying capacity of short sequences, and therefore, it is recommended to input sequences with more than 40 AAs in GIVAL for prediction. The prediction performance of GIVAL was further validated with sequences from several influenza pandemics, and a cross-family generalization test was conducted by predicting influenza B virus (IBV) with a model trained on IAVs (Fig. S17A). All of the IAV sequences from the 2009 H1N1 and 1968 H3N2 pandemics and the IBVs were predicted to be human-adaptive, confirming the rationality of GIVAL’s prediction (Fig. S17B). Therefore, the GIVAL framework provides a solution for the generalized adaptation prediction of input virus sequence with a varied length.

### GIVAL predicts and infers high-risk mammalian H3N8 and bovine H5N1 IAVs

**Fig. 5.**
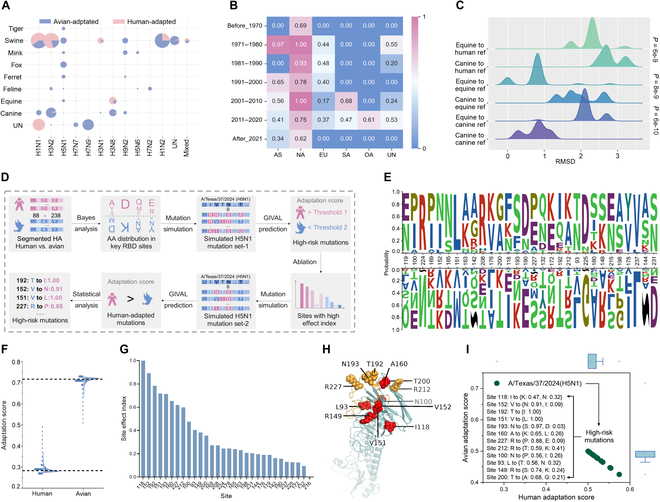
Prediction and inference of human-adapted H3N8 and H5N1 IAVs based on HA RBD. The ratio of human- or avian-adaptive samples of each serotype and host (A) and the HRI of samples collected in each time period and continent (B) (AS, NA, EU, SA, OA, and UN represent Asia, North America, Europe, South America, Oceania, and unknown, respectively). (C) The RMSD between the HA RBD structure of H3N8 reference sequences and other human-adaptive equine H3N8 and avian-adaptive canine H3N8 based on GIVAL prediction. (D) The pipeline of high-risk H5N1 mutations prediction and quantification. (E) Logo plot of the AA distribution of the top 30 important sites in human- and avian-adaptive HA RBD sequences. (F) The predicted human and avian adaptation score for the 30,000 mutations according to AA distribution. The dashed lines represent the score of the reference H5N1 strain [A/Texas/37/2024(H5N1)]. (G) Effect indexes of top 30 sites. (H) The distribution of the top 12 sites (colored in red) and the 130-loop, 190-helix, and 220-loop (colored in orange) on the protein structure of the reference strain. (I) The predicted adaptation score of the human-adapted mutations with only the top 12 sites mutated.

Initially, GIVAL in response to the input of IAV HA RBD was applied to predict the adaptation of mammalian IAVs other than human IAVs. Firstly, the host preference with spatiotemporal distribution was obtained. More than 80% of H3N2/H3N1, 59% of H1N1, and 25% of H1N2 were predicted to be human-adaptive for the swine IAV HA samples, whereas most HAs from other mammals were predicted to be avian-adaptive (Fig. 5A). Surprisingly, more than 83% of equine H3N8 HAs were predicted to be human-adaptive (Fig. 5A). Spatiotemporal analysis of these predicted samples indicated a continuous high adaptation of the IAV HAs from North America to humans and sporadic high adaptation in Asia from 1971 to 1980, in South America from 2001 to 2010, and in Oceania from 2011 to 2020 (Fig. 5B). Secondly, a Bayes method was utilized to screen key AAs and their locations in HA for H3N2 and H3N8, respectively, to biologically interpret the predicted host preference. A marked difference in AA distribution was observed at sites 134, 132, 186, 223, 193, 183, 190, or 226 between human-adaptive and avian-adaptive H3N2 HA sequences predicted by GIVAL. These sites were located near the RBD, which included the 130-loop, 190-helix, and 220-loop (Fig. S18A). A high frequency of Leu at the 219 site, Val at 115, Asp at 101, Ala at 135, and Asn at 89 and 185 was shown for predicted avian-adaptive H3N8 HAs, compared to the high frequency of Trp at 219, Leu at 115, Asn at 101, Ser at 135 and 89, and Thr at 185 for predicted human-adaptive H3N8 HAs (Fig. S18B). Thirdly, the predicted host preference of H3 samples was verified using protein structure prediction. The root mean square deviation (RMSD) value showed that the structure of the swine H3N2 samples, which were predicted to be human-adaptive, was significantly different from that of the canine H3N2 samples, which were predicted to be avian-adaptive and were significantly closer to human H3 (*P* < 0.0001, Fig. S18C), and the structure of equine H3N8 samples, which were predicted to be human-adaptive, was significantly different from that of canine H3N8 samples, which were predicted to be avian-adaptive and were also significantly closer to human H3 (*P* < 0.0001, Fig. 5C). The HA RBD sequences that were predicted to be human- or avian-adaptive differed at sites 132, 213, and 220 of canine H3N8 (Fig. S18D) and sites 135, 141, and 220 of equine H3N8 (Fig. S18E). To validate our predictions regarding mammalian adaptation, we characterized the receptor-binding properties of IAV HA1 proteins using bio-layer interferometry (BLI) with α2,3-linked (3′-SLNLN) and α2,6-linked (6′-SLNLN) sialylglycan receptors. Initial validation using avian H5N1 (Fig. S19A and B) and human H3N2 (Fig. S19C and D) HA1 proteins as control successfully reproduced their characteristic binding profiles, with human H3N2 exhibiting stronger binding ability for 6′-SLNLN and avian H5N1 HA1 showing preferential binding to 3′-SLNLN, thereby confirming the reliability of our experimental system. Importantly, equine H3N8 exhibited stronger binding to 6′-SLNLN than to 3′-SLNLN (Fig. S19E and F), consistent with the predicted potential for human adaptation in equine H3N8 HA RBD.

Additionally, high-risk H5N1 mutations for humans were inferred using GIVAL based on the HA RBD model (Fig. 5D). Firstly, the top 30 sites with relatively high importance values and clear differences between the AA distribution of human- and avian-adaptive sequences (Fig. 5E) were selected. Secondly, the adaptation of the mutations generated with the top 30 sites mutated were predicted and quantified using GIVAL. Compared with the reference strain, the overall avian adaptation score decreased and the human adaptation score increased (Fig. 5F). Thirdly, according to the downward trend of the site effect indexes of the 30 sites (Fig. 5G), the top 12 and top 8 sites were respectively selected for generating mutations, and the selected 12 sites were found to be close to or within the HA 130-loop, 190-helix, and 220-loop on the predicted protein structure of the reference strain (Fig. 5H). According to GIVAL prediction, 34 mutations with 12 sites (Table S3) mutated and 2 with 8 sites (Table S4) mutated were predicted to be human-adaptive, and V152N, T192I, V151L, and N193S mutations on H5N1 HA might largely affect the adaptation risk (Fig. 5I). The relationship between the high-risk H5N1 mutations and the circulating H5N1 strains was analyzed, and it was found that a large proportion of circulating strains containing the high-risk mutations were from dairy cows in North America and might pose a potential threat to transmission in human population (Table S5).

To summarize, a large proportion of equine H3N8 IAVs were predicted to be human-adaptive, supported by protein structure prediction, and high-risk H5N1 mutations were inferred using GIVAL.

### GIVAL predicts marked adaptation shift of the prevalent monkeypox viruses

**Fig. 6.**
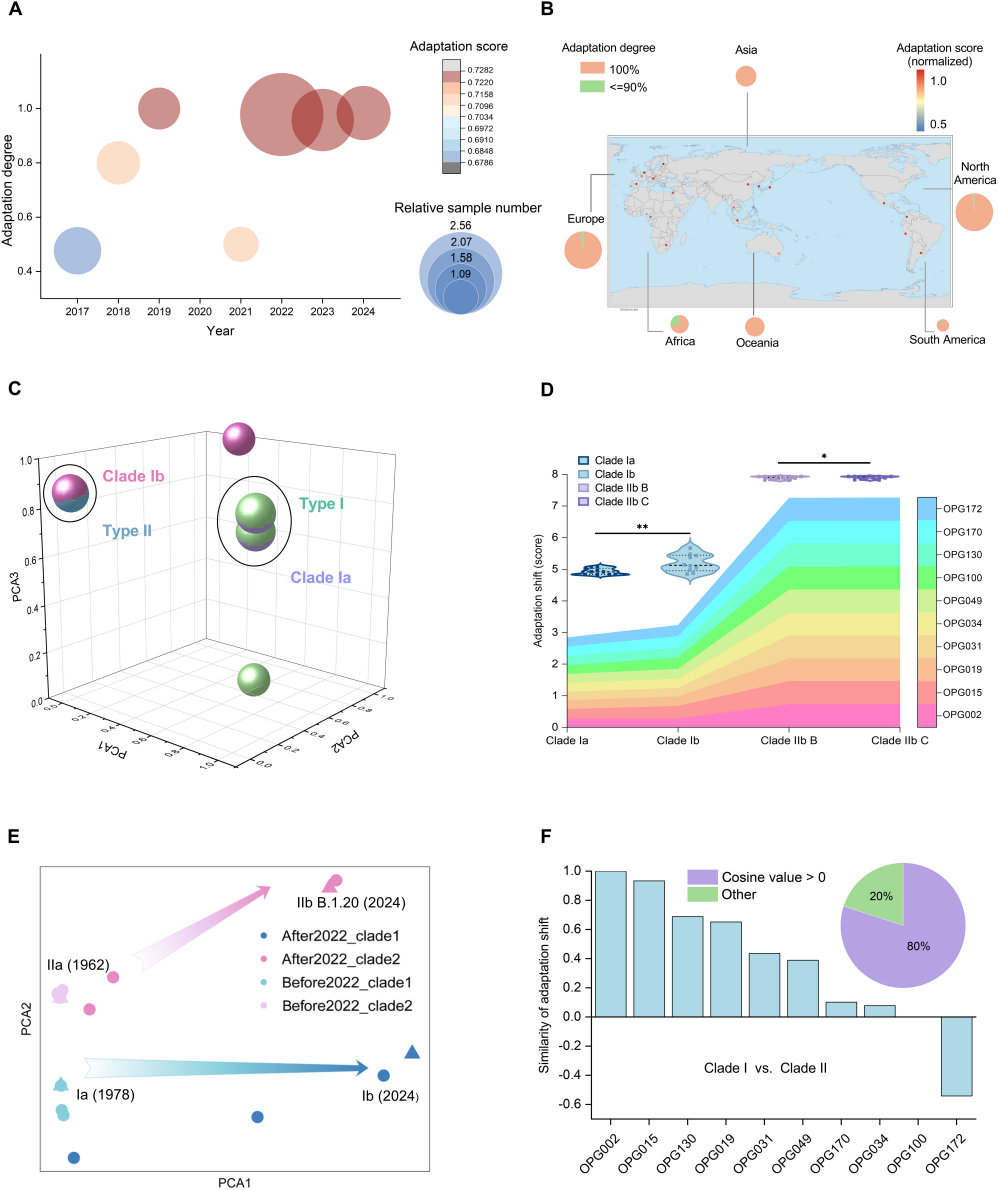
Adaptation shift prediction of monkeypox viruses based on main proteins. Mean adaptation degree and score of each year (A) and region (the official review number of the map is “GS(2016)1665”). (B) of 10 proteins of monkeypox viruses post-normalization (to a range of 0.5 to 1.0). (C) Reduced 3 components with PCA of FC vector of *OPG015* sequences from types I and II in the training set and clades Ia and Ib in the validation set. (D) The average adaptation score of 10 proteins of clades Ia, Ib, IIb B, and IIb C. (E) Reduced 2 components with PCA of vBERT-embedded *OPG015* sequences from clades I and II collected before and after 2022. The adaptation shift from clade IIa to IIb B.1.20 and that from clade Ia to Ib were emphasized with arrows [triangles represent the 4 representative sequences, namely, IIb B.1.20 (2024): EPI_ISL_19459746; IIa (1962): EPI_ISL_13056556; Ib (2024): EPI_ISL_19345034; Ia (1978): EPI_ISL_13058456, and circles represent other sequences]. (F) The angle cosine between adaptation shift vectors of clades I and II of each protein.

The adaptation shift of the prevalent monkeypox viruses was predicted using GIVAL. Firstly, according to the score of vBERT embedding (Fig. S20A), DNT (Fig. S20B), and other prior knowledge, *OPG002*, *OPG015*, *OPG019*, *OPG031*, *OPG034*, *OPG049*, *OPG100*, *OPG130*, *OPG170*, and *OPG172* were respectively selected for adaptation prediction. Secondly, the prediction performance was validated with high accuracy of the model based on each protein (Fig. S21A to J). Thirdly, the spatiotemporal distribution of the adaptation shift was obtained. A higher reported number and score for type II adaptation probability from 2022 to 2024 was shown, which might reflect a higher transmission risk of the adaptation shift in the future (Fig. 6A). A higher transmission probability was observed in North America and Europe, and sequences of the predicted dataset in Asia and Oceania were found to be completely type II adaptive. A relatively high portion of samples in Africa showed partial type I adaptability, which indicated that part of these samples might have higher pathogenicity (Fig. 6B). Additionally, the relationship and similarity between the adaptation shift of each clade was further analyzed. A shorter fully connected layer (FC)-based distance was observed from monkeypox viruses with type II adaptation to clade Ib than to clade Ia in most of the proteins (Fig. 6C and Fig. S22A to J), which was consistent with the incremental type II adaptation shift from clade Ia to Ib and from IIb B to IIb C (Fig. 6D). Most monkeypox viruses circulating before 2022 were found to be clade Ia, IIa, and IIb A, while samples after 2022 were mainly from clade Ia, Ib, IIb B, and IIb C (Fig. S23). Similarities between the adaptation shift from clade Ia before 2022 to clade Ib after 2022 and from clade IIa before 2022 to clade IIb B after 2022 were indicated in 80% of the proteins based on the vBERT-optimized embedding (Fig. 6E and F and Fig. S24A to J). Generally, a distinct incremental adaptation shift has been predicted using GIVAL for monkeypox viruses since 2022.

## Discussion

It is challenging to accurately assess the human adaptation of an emerging virus in advance, based solely on its genotype. Intelligent genotype-to-phenotype prediction has provided a promising risk assessment strategy for such cases. However, there is still a generalization insufficiency for currently available artificial intelligence predictors, which are inflexible after training and are limited to a specified virus protein within a narrow length range. This insufficiency was addressed in the present study with a general intelligent framework, integrating multiple pipelines of input virus protein sequence retrieval, sequence embedding with a pretrained language model, input-mapped semi-supervised learning, and statistical inference of adaptation based on the identical distribution of the predicted sample with some group of adaptation-labeled samples.

The protein language model of vBERT for all viruses outperformed the accurate embedding multi-grained genotype–phenotype association. The best performance of vBERT was achieved on embedding by balancing data size and genome variation with statistical sampling and a more context-dependent tokenization of the viral protein sequence using NLP techniques. vBERT was competent at multiple-grained embedding tasks, such as coarse-grained protein clustering and fine-grained key mutation capture, implying a deep understanding of the biological semantics. Moreover, the GIVAL framework can be generally utilized for tasks, such as viral adaptation phenotype assessment based on an unspecified “X” viral protein sequence with full or segmented length. Dataset mapping and labeling, model training, and risk prediction were automatically performed upon input into GIVAL. The “X” input was specifically mapped to its viral protein sequence in a database of currently available virus reference genes via the benchmarked mapping tool of BLAST+ before training an input-specific predictor. Mismatch of adaptation labels for some sample records in public databases was another defect in training a virus adaptation model, owing to biologically unreasonable labeling of adaptation hosts. For example, H5N1, H7N9, and some other IAV samples in public databases were labeled as human hosts, although they were untransmissible in the human population after spillover infection [42,43]. Such a defect could be partially remedied based on the data distribution of vBERT-embedded sequences [44] and, thus, also contributed to the outperformance of GIVAL in predicting the host adaptation of the virus. The data distribution-based label setting was also more reasonable than the arbitrary setting of label numbers, followed by the statistical inference of the adaptation of the input. The inference of adaptation based on predicted flexible labels effectively addresses the limitations of incomplete raw adaptation-related annotations in datasets, enabling comprehensive analysis of various adaptation annotations for input sequences. Notably, influenza viruses exhibit unique characteristics where sequences sharing the same adaptive host label may belong to diverse serotypes, resulting in relatively high heterogeneity among sequences within each flexible label category. To overcome the limitations and potential inaccuracies of direct inference using the most frequent adaptation label from predicted flexible labels, we developed a hierarchical algorithm for determining adaptation thresholds. For predicting sequences of other viruses, users may either employ our default method or optionally utilize this hierarchical approach for adaptation analysis, depending on their specific research needs. The performance of GIVAL was validated by accurately predicting the host-specified receptor binding of IAVs and CoVs.

The trained GIVAL model upon an input of RBD-containing HA segment was utilized to predict the adaptation risk of IAVs that were identified in avian or mammalian hosts other than humans in recent years, since it is never excessive to worry about the next influenza pandemic. Firstly, a high human adaptation of swine H3N2/H3N1 or swine H1N1 IAVs was predicted by GIVAL, which was consistent with the well-known human receptor-binding specificity [45,46]. Surprisingly, a discrepancy in the adaptation of receptor binding was observed based on GIVAL predictions between equine and canine H3N8 IAVs. More than 75% of equine H3N8 HA were predicted to be human-adapted, whereas canine H3N8 HA was not predicted by GIVAL to adapt to humans, which was proven by the smaller difference in the structural RMSD values between the HA RBD of equine H3N8 to human H3 than that between canine H3N8 and human H3. The potential for cross-species transmission of equine H3N8 to humans also verified the above predictions [47].

The currently prevalent H5N1 spread in birds and mammals has raised concerns about another influenza pandemic [48], although only limited binding of it to human receptors has been observed [49]. It is more urgent to warn of possible mutations with increasing human receptor binding. GIVAL predicted some potential variants with mutations within or near HA RBD, such as T192I, V151L, and R227P. We propose that in-time and close attention should be paid to these possible mutations and the adaptation shift of receptor binding of present bovine H5N1 viruses.

The monkeypox virus poses another concern for pandemics [50]. In particular, there was a sharp increase in the number of cases and a spread of monkeypox worldwide. GIVAL automatically provided 2 types of adaptation labels based on data distribution, consistent with the virological classification of type I with higher morbidity and mortality and type II with higher transmission ability [51], based on 10 main viral proteins. According to the risk index of qualitative adaptation degree and quantitative score, the major circulating monkeypox viruses have undergone a marked shift in human adaptation after 2022. High type II adaptation risk was also found in viruses from some regions in North America, Asia, Europe, and Oceania, based on test data from the National Center for Biotechnology Information (NCBI), which is consistent with the endemic regions over the past decades [52]. Additionally, there is a similar adaptation shift of clade Ia to Ib and of clade IIa to IIb B/IIb C, consistent with the evolution of the adaptation pattern [53,54].

However, there is much performance space to update the embedding tool of vBERT and the GIVAL prediction framework. It is of great importance for establishing a language model based on a sufficiently rich and balanced virus protein data space. However, the unbalanced availability and distribution of virus samples has limited the effectiveness of vBERT embedding, although statistical sampling was conducted for vBERT to balance the number of positive and negative samples [55]. In our study, the dataset for vBERT pretraining was also statistically downsampled based on biological knowledge. However, oversampling was not performed to prevent data distortion. Therefore, it is crucial to further optimize the balanced number of sequences without affecting the dataset’s structure. Reasonable labeling is the key to training a supervised learning prediction model. A semi-supervised model utilizes unlabeled data more effectively by learning the probability distribution and classes [56]. In our study, the semi-supervised framework overcomes the label insufficiency of recorded sequences in public databases and is thus more applicable for phenotype prediction tasks based on sequences with insufficient labels. However, owing to the lack of information on virus families and names of viral proteins for some recorded samples, the mapped dataset was extracted based on the homology between sequences, which may lead to low accuracy of the prediction for some virus families. The imbalance in the number of various viruses has led to imperfect flexible labeling, resulting in difficulties in predicting some phenotypes. Summarily, how to overcome these limitations to achieve more accurate predictions on more enriched tasks is a vital issue to be explored.

In summary, GIVAL provides a framework for automatic sample labeling, data sampling, and model training to obtain integrated adaptation outputs upon an “X” viral protein input. GIVAL can not only assess the adaptation risk of currently prevalent viruses, such as IAVs and monkeypox viruses, but also predict possible high-risk variations of the virus in concern, such as bovine H5N1 viruses.

## Materials and Methods

### Pipeline of GIVAL for viral protein embedding and adaptation prediction

Viral protein sequences from 42 families were parsed, tokenized using HMM, and segmented for pretraining the viral protein embedder, vBERT. The optimized vBERT was selected through parameter optimization and benchmarking against other pre-trained models. Leveraging vBERT embeddings, the semi-supervised GIVAL framework was developed for virus and protein identification, dataset label optimization, and adaptive risk prediction, with risk quantification performed using a timely trained ResNet model.

### Preparation of viral protein sequences

Sequences of viruses downloaded from NCBI (https://www.ncbi.nlm.nih.gov/nuccore/), Global Initiative of Sharing All Influenza Data (GISAID) (https://gisaid.org/), and Bacterial and Viral Bioinformatics Resource Center (BV-BRC) (https://www.bv-brc.org/) were cleaned and protein sequences (with more than 50 amino acids) were extracted. Sequence deduplication was performed to create the whole dataset (1.86 million protein sequences from 42 families). Strain names and other annotations were extracted using Python scripts. Sampling was conducted based on different methods for different types of virus sequences in the deduplicated whole dataset. Protein sequences from the faa file were firstly sampled to ensure that the number of sequences in each family did not exceed 6,000. The number of sequences from the gb and faa files were then sampled to 147,850 based on the homology between the sequences. The number of SARS-CoV-2 sequences were sampled to 20,000 according to the Pango lineage. Host-based sampling was conducted to IAV sequences to ensure that the number of sequences from each host did not exceed 6,193 and IAV sequences were sampled to 19,999. Sampling was not performed in 9,213 non-SARS-CoV-2 CoV sequences to balance the number of sequences from different types of viruses. The sampled dataset (197,062 sequences) was utilized for the HMM tokenizer and 100,000 sequences were further randomly sampled for vBERT pretraining. Additionally, the sampled dataset with 100,000 sequences and with 50,000 sampled sequences of IAV HA added was created as the simulated dataset. The Simpson index and normalized Simpson index were selected to analyze the diversity of sequences in the datasets. The indexes were calculated using Eqs. 1 and 2:

$$\text{Simpson index}=1-\sum_{i=1}^{S}p_{i}^{2}$$ (1)

$$\begin{array}{c} \text{Normalized Simpson index}=\left(1-\sum_{i=1}^{S}p_{i}^{2}\right)/\left(1-\frac{1}{S}\right) \end{array}$$ (2)

In Eqs. 1 and 2, $p_{i}$ represents the frequency of the ith type of sequence, and S represents the number of types.

### Pretraining and benchmarking of vBERT for viral protein embedding

#### Tokenization and segmentation of viral proteins

Flexible tokenization was performed with HMM, and specified tokenization with a fixed number of AAs as tokens and BPE tokenization were performed as a control. The HMM and BPE tokenizer (trained with the Python package tokenizers.trainers.BpeTrainer) were trained on the HMM dataset.

To establish the HMM, the Python package jieba was downloaded from GitHub (https://github.com/fxsjy/jieba/). Sequences were firstly tokenized with 1 to 5 AAs as tokens, and vocabulary selection was performed. Based on the selected vocabulary list, the sequences were firstly tokenized without HMM. The statuses of AAs can be divided into 4 categories, namely, beginning (B), ending (E), middle (M), and single (S). For tokens spanning multiple AAs, B denoted the first position, E the last position, and M all intermediate positions; for single-AA tokens, the position was labeled as S. On this basis, the starting, emit, and transition probabilities of 20 types of amino acids and one unknown character were calculated to establish HMM using Eqs. 3 to 5:

$$\text{Starting probablity}\;:P_{k}=P\left(X_{1}=S_{k}\right)$$ (3)

$$\text{Emit probablity}\;:P_{k}\left(b\right)=P\left(y_{i}=b|X_{i}=S_{k}\right)$$ (4)

$$\text{Transmission probablity}\;:P_{\mathrm{kl}}=P\left(X_{t+1}=S_{l}|X_{t}=S_{k}\right)$$ (5)

In Eqs. 3 to 5, $\;X_{i}$ represents the status of the ith AA, $S_{i}$ represents the ith status, $y_{i}$ represents the ith AA, and b represents the AA of interest. Using these probabilities, the Viterbi algorithm in HMM determined each AA token boundary. Viral protein tokenization with HMM and sequence segmentation from random start locations based on the maximum token limit of vBERT were performed. The tokenized vocabularies were deduplicated to create a pretraining vocabulary list.

To further evaluate the performance of the HMM tokenizer, the vocabulary frequency vectors of each type of RNA or DNA virus were counted, and the connection index (CI) was defined using Eq. 6:

$${\mathrm{CI}}_{i,j}=1-\text{cos}\left(v_{i},v_{j}\right)$$ (6)

In Eq. 6, $v_{i}$ and $v_{j}\;$represent the vocabulary frequency vectors of the ith and jth RNA/DNA family. The frequency of 20 AAs and an unknown character in each sequence of the sampled dataset (real dataset) was counted. Virtual sequences, equal in number, were generated based on the AA frequency vectors to form a virtual dataset to further evaluate the HMM performance. More details about the performance evaluation of HMM can be found in the “Performance evaluation of HMM tokenizer” section in the Supplementary Materials.

#### Pretraining of vBERT embedder and parameter optimization

To pretrain the vBERT model for viral protein embedding, a BERT-base configuration (12 encoder layers, 12 attention heads, 163M parameters, and 768 hidden size) was chosen, with a training batch size of 32, a learning rate of 2e−4, and AdamWeightDecay optimization with a $\beta_{1}$ of 0.900 and a $\beta_{2}$ of 0.999. The total number of tokens in the sampled dataset with 100,000 sequences and the whole dataset were 37 million and 1 billion, respectively. Sequences were segmented into segments with fewer than 256 tokens. vBERT pretraining code was from the original BERT model (https://github.com/google-research/bert/), using the [CLS] token and summation of token, segment, and position embeddings, with “max position embeddings” set to 512.

For parameter optimization, vBERT models were pretrained on various datasets (sampled 100,000, whole, simulated) with different tokenization (HMM, BPE, and fixed-AAs [2-AA, 3-AA, and 4-AA]), segmentation (no segmentation, 96-token, and 256-token), learning rates (2e−3, 2e−4, and 2e−5), and training steps (220,000, 300,000, and 380,000) based on BERT. To study the impact of model size, BERT-tiny (2 encoder layers, 2 heads, hidden size 128) and BERT-medium (8 layers, 8 heads, hidden size 512) were selected to pretrain another 2 vBERT models. Fixed-AAs vBERT models were set as ablation to compare with HMM vBERT to quantify the HMM tokenization impact.

#### Benchmarking of vBERT embedding for viral proteins

For each IAV serotype or SARS-CoV-2 type, 200 sequences (2,000 for HA and 1,000 for Spike RBD) were randomly sampled to form the test dataset. The Transformer [57], ESM-2 [58], DNABERT-2 (https://arxiv.org/abs/2306.15006), and proteinBERT [59] were selected as benchmarking models. The reduced 2 components with t-distributed stochastic neighbor embedding [60] of vBERT-embedded and padded sequences of the 2 datasets were clustered. More details on dimensionality reduction and clustering can be found in the “Dimensionality reduction and clustering of embedded sequences” section in the Supplementary Materials. The embedding performance of each model was evaluated using 5 clustering indexes, namely, silhouette score (silhouette), Calinski-Harbasz Score (CH), Davies–Bouldin index (DBI), Adjusted Rand Index (ARI), and Normalized Mutual Information (NMI). The vBERT model that obtained more optimal indexes was selected. Statistical testing was conducted based on the methods described in the “Statistical testing of significant differences” section in the Supplementary Materials.

The IAV NP dataset (500 human-adaptive and 500 avian-adaptive sequences) was sampled to evaluate the embedding performance at each key site. One key site was selected each time, and token embeddings including the site were extracted from vBERT and Word2Vec. All tokens in the sequences were clustered into 5 categories for each model. The maximum proportion of embeddings of tokens including each selected site in the same cluster based on the embeddings of the 2 models was calculated and compared. Additionally, vBERT embedding performance was evaluated on immune escape assessment and DMS datasets, and more details can be found in the “vBERT embedding-based immune escape analysis of IAV vaccine strains” and “vBERT embedding-based mutational effect analysis of single amino acid” sections in the Supplementary Materials.

### Establishment and evaluation of GIVAL to predict virus adaptation

#### Mapping input “X” sequence to viral protein dataset

All available virus reference sequences (9,231 records) were downloaded from the NCBI database. After cleaning, 98,939 protein sequences were extracted for mapping. The whole dataset (1.86 million sequences) was selected as the sequence dataset for GIVAL prediction, and users can add other virus and reference sequences for subsequent steps. To improve efficiency, a smaller-scale reference dataset (742 sequences) and sequence dataset (26 101 sequences) were also provided.

The input protein sequence segment was mapped to the reference dataset for sequence retrieval using customized BLAST+ (BLAST+ 2.16.0 with the reference dataset as the database), and the mapping results were further verified. The customized BLAST+ based on our reference dataset was selected to keep the consistency between the annotations of the identified viral gene and the viral gene in the sequence dataset, so as to guarantee the correct matching of the dataset related to the input sequence. Details on dataset retrieval can be found in the “Dataset retrieval in GIVAL” section in the Supplementary Materials.

To map with the retrieved protein dataset, if it was already aligned, sequences in the retrieved dataset were cut according to the reference sequence mapping location. Otherwise, the first 20 amino acids of the mapped segment were aligned with each dataset sequence via a sliding window, and the best mapping positions were located by identifying the domain with the lowest Levenshtein distance (LD). The last 20 amino acids were located using Eqs. 7 to 9:

$$l_{e0}=l_{s}+\text{length}\left(\text{segment}\right)-1,$$ (7)

$$LD_{k,l}≝\mathrm{LD}\left(\mathrm{end}\;20\;\mathrm{AAs},\;{\mathrm{ms}}_{k,l}\right),$$ (8)

le=le0,ifLDle0−19,le0<6le0+argminx∈−55LDle0−19+x,le0+x,else. (9)

In Eqs. 7 to 9, $l_{s}$ and $l_{e}$ represent the starting and ending sites of the mapped domain on the sequence in the matched dataset, respectively, and ${\mathrm{ms}}_{i,j}$ represents site i to site j of the mapped sequence (sites i and j were included).

#### Establishment of semi-supervised GIVAL based on vBERT embedding

Semi-supervised learning was conducted for the integrated prediction of the input sequence. Firstly, the optimized labels were obtained via unsupervised learning based on the vBERT embedding. The input sequence and the retrieved dataset were embedded with vBERT. The embedded retrieved dataset was reduced using PCA and clustered to obtain flexible labels. Random sampling was performed to balance the sequence numbers from each flexible clustering label to create the training dataset.

Secondly, the supervised ResNet predictor was trained based on the flexible labels. Each sequence embedding in the training dataset was padded to ensure that the size of the input embedding was a fixed integer multiple of 16×768. The embeddings were reshaped into $\left(c,64,64\right)$ as the ResNet input (c positive integer). The ResNet model was built with a 64-channel convolution neural network, a 64-channel max pooling layer, residual blocks (3, 4, 6, and 3 layers with 64, 128, 256, and 512 channels, respectively), average pooling, and a dropout layer. BatchNorm normalization and ReLU activation were selected. The input was converted into $\left(64,32,32\right)$ after convolution, $\left(64,16,16\right)$ after max pooling, and $\left(64,16,16\right)$, $\left(128,8,8\right)$, $\left(256,4,4\right)$, $\left(512,2,2\right)$ after each residual block calculation, and then $\left(512,1,1\right)$, and original probabilities of flexible labels were predicted and output with Softmax function. The dimensionality of the output layer in the real-time trained model is dynamically adjusted based on the input, equaling the number of flexible cluster labels present in the training dataset. The dataset was split into 3 training–validation datasets for 3-fold cross-validation. Training was stopped when both the training and validation accuracies exceeded the threshold.

Finally, the flexible label of the input sequence was predicted using the timely trained ResNet model, and the adaptation of the input sequence was further statistically inferred based on the predicted flexible label and the raw annotations related to viral adaptation in the training dataset. During the training of the prediction model, we relied solely on sequence distribution-based flexible labels rather than the raw annotations from the dataset. This allows users to analyze different types of adaptation annotations according to their specific needs when utilizing the model. In this study, we primarily examined annotations related to influenza virus host adaptation, CoV receptor-binding specificity, and monkeypox virus pathogenicity and transmissibility in humans. Our model employs a hierarchical approach to determine input sequence adaptation, with the “adaptation threshold” defined as follows. Adaptation labels were determined by the majority of adaptive hosts or type annotations in the predicted flexible cluster for non-influenza viruses. Based on the FC vector, the adaptation risk was further quantified using Eqs. 10 to 13:

$$CD\left(FC_{x},type\;i\right)=\underset{m_{i}}{\text{min}}\left(\frac{1}{n_{m_{i}}}\sum_{j=1}^{n_{m_{i}}}\left(1-\text{cos}\left(FC_{x},FC_{y_{j}}\right)\right)\right),$$ (10)

$$CD^{\prime }{\text{score(FC}}_{x},\text{type i)}=1-\frac{CD\left(FC_{x},\text{type}\;i\right)}{\sum_{i}CD\left(FC_{x},\text{type}\;i\right)},$$ (11)

$$\begin{array}{c} \begin{array}{c} \text{Adaptation scor}e_{i}=\text{Softmax}\left(CD^{\prime }\text{score}\left(FC_{x},\text{type}\;i\right)\right),\\ \text{If Adaptation scor}e_{\text{predicted label}}\neq \text{maximum score}, \end{array} \end{array}$$ (12)

$$\text{Adaptation scor}e_{i}=\left\{\begin{array}{c} \frac{1}{n}+0.0001,\text{if}\;i=\text{predicted label}\\ \frac{1-\left(\frac{1}{n}+0.0001\right)}{n-1},\text{else} \end{array}\right..$$ (13)

In Eqs. 10 to 13, $m_{i}$ represents the flexible label with type i adaptation, $n_{m_{i}}$ represents the sample number of flexible label $m_{i}$, x represents the predicting sample, $y_{j}$ represents sample in the training set with flexible label $m_{i}$, and n represents the number of adaptation labels.

Given the high diversity of influenza virus subtypes from each adaptive host, the adaptation labels of the input were inferred according to the following principles if the virus was identified as influenza virus. If the majority host count in the predicted flexible cluster was at least 3 times greater than that of any other host label, the adaptation label was inferred using the above-mentioned method; otherwise, the host adaptation label was determined based on Eqs. 14 and 15:

$$CD\left(FC_{x},\text{type}\;i\right)=\underset{k\in K_{i}\;}{\text{min}}(1-\text{cos}\left(FC_{x},FC_{k}\right)),$$ (14)

$${\text{Adaptation label}}_{x}=\underset{i\;}{\text{argmin}}(CD\left(FC_{x},\text{type}\;i\right)).$$ (15)

In Eqs. 14 and 15, $K_{i}$ represents the sequences with adaptation label i in the predicted flexible cluster of x. ${\text{Adaptation label}}_{x}$ represents the inferred adaptation label of the input.

#### Performance evaluation of GIVAL for clustering and classification

Firstly, the mapping performance was evaluated and benchmarked. A test dataset for mapping was created by randomly extracting 500 segments with a length of 150 to 300 AAs from the sequence dataset for GIVAL prediction. The customized BLAST+ was benchmarked with Diamond [61] on the test dataset based on the mapping accuracy. The IAV HA RBD segment (151 AAs from sites 88 to 238, excluding the signal peptide of NC_007362.1) and SARS-CoV-2 Spike RBD segment (207 AAs from sites 344 to 550 of NC_045512.2) were mapped using the customized BLAST+ and Diamond, and the results were compared by extracting the mapping identity of the input segment and the mapped reference sequences. Notably, all sites on HA sequences in this study were renumbered by offsetting the 12-residue signal peptide to maintain consistent positional references.

Secondly, complete HA sequences were selected to establish GIVAL for flexible labeling and GIVAL performance evaluation. Flexible and specified labeling based on host labels were conducted for ablation to quantify the contribution of flexible labeling. Human-adaptive H3 sequences highly homologous to clade 3c.3a [62,63] and avian-adaptive H5 sequences were extracted from the training dataset (2,343 sequences) to create an independent validation set (657 sequences). The silhouettes of flexible and specified clusters were calculated and compared. To further evaluate the impact of flexible labeling on prediction, while training models based on 2 types of labels, 5%, 10%, 15%, and 20% of the human-adaptive H3 sequences in the training dataset were mislabeled as avian-adaptive to train the prediction models. The performance of the models on the independent validation dataset was compared using 5 indexes, namely, Accuracy (ACC), Precision of human-adaptive (Pre_P) and avian-adaptive (Pre_N) cluster, and Recall of human-adaptive (TPR) and avian-adaptive (TNR). The indexes were calculated using Eqs. 16 to 20:

$$\mathrm{ACC}=\frac{\mathrm{TN}+\mathrm{TP}}{\mathrm{TN}+\mathrm{TP}+\mathrm{FN}+\mathrm{FP}},$$ (16)

$$\mathrm{Pre}\_P=\frac{\mathrm{TP}}{\mathrm{TP}+\mathrm{FP}},$$ (17)

$$\mathrm{Pre}\_N=\frac{\mathrm{TN}}{\mathrm{TN}+\mathrm{FN}},$$ (18)

$$\mathrm{TPR}=\frac{\mathrm{TP}}{\mathrm{TP}+\mathrm{FN}},$$ (19)

$$\mathrm{TNR}=\frac{\mathrm{TN}}{\mathrm{TN}+\mathrm{FP}}$$ (20)

Thirdly, the HA RBD and Spike RBD segment were respectively input to establish GIVAL for performance evaluation. The prediction accuracy of the 2 models was tested based on confusion matrices and ROC with AUC. The flexible labels of the mapped CoV Spike dataset (970 sequences) were verified by constructing maximum-likelihood phylogenetic trees using MEGA and iTOL (https://itol.embl.de/). Based on the CoV Spike RBD model, the PDF-2180 (NC_034440.1) and NeoCoV (KC869678.4) were predicted and verified. On this basis, the prediction robustness for extreme sequence lengths was evaluated, and adaptation risk of IAVs from pandemics and IBVs were predicted for validation and cross-species generalization test of GIVAL. The related details can be found in the “Prediction robustness evaluation of GIVAL for extreme sequence lengths” and “Validation and cross-species generalization test of GIVAL based on IAV from pandemics and IBVs” sections in the Supplementary Materials.

### Risk assessment of multiple viruses via vBERT embedding and GIVAL prediction

#### Workflow for applying GIVAL to real-world viral adaptation scenarios

For an unknown viral protein sequence input, GIVAL follows the pipeline below for viral and gene identification, dataset matching, label optimization, model training, and adaptive risk prediction. Firstly, the reference sequences were utilized to establish the reference database for BLAST+ mapping, and the virus and gene of the input were identified. Secondly, the mapping result was extracted to match the proper dataset for training the predictor. The label of each sequence in the matched dataset was optimized based on vBERT embedding. Thirdly, based on the training dataset with optimized labels, a ResNet model was trained to predict the optimized label of the input. Finally, the adaptation risk was statistically inferred. Based on existing databases, users can add new sequences to both the reference dataset and the sequence dataset as needed. The identified virus and gene, information of the optimized labels, trained predictor, predicted optimized labels, and inferred adaptation risk were automatically saved.

#### Adaptation risk prediction of multiple viruses

Firstly, based on the trained IAV HA RBD prediction model, the adaptation risk of mammalian IAVs and H5N1 mutations was predicted. The adaptation of HA RBD sequences of mammalian IAVs (5,403 sequences) was predicted and verified using protein structure prediction, Bayesian inference, and experimental validation. More details on protein structure, Bayes inference, mammalian IAV prediction and experimental validation of the predicted adaptation of equine H3N8 IAVs can be found in the “Protein structure prediction, alignment and visualization”, “Bayes inference of adaptation-important amino acid sites”, “Adaptation prediction of IAV HA RBD from mammalian hosts”, and “Binding kinetics analysis of HA1-Glycan interactions by bio-layer interferometry (BLI)” sections in the Supplementary Materials. Based on the key sites and AA distribution obtained by Bayesian analysis, 30,000 H5N1 HA RBD mutations were generated and high-risk mutations were inferred. More details on high-risk IAV H5N1 mutation inference can be found in the “Adaptation prediction and quantify of H5N1 mutations” section in the Supplementary Materials.

Secondly, the monkeypox virus adaptation shift was predicted using GIVAL with 10 proteins. The adaptation shift was predicted using samples from clade I before 2022 and clade II before 2024 as the training set (300 sequences per protein) and clade I after 2022 (including 2022) and clade II in 2024 as the validation set (318 sequences per protein). More details on the prediction of monkeypox virus adaptation shift can be found in the “Prediction of adaptation shift for monkeypox viruses” section in the Supplementary Materials.

## Data Availability

Code and data were available online (https://github.com/Jamalijama/GIVAL and https://doi.org/10.5281/zenodo.16566992) or upon request (J.L., lj-pbs@163.com).
