# Supplementary material for: General Intelligence Framework to Predict Virus Adaptation Based on a Genome Language Model
Source: Research (Wash D C). 2025 Sep 30;8:0871. doi: 10.34133/research.0871 (PMC12480747; doi:10.34133/research.0871)
Supplement: Supplementary 1 — Supplementary Methods Figs. S1 to S24 Tables S1 to S5 [file research.0871.f1.pdf]

General Intelligence Framework to Predict Virus Adaptation based on a Genome Language Model

Running title: **GIVAL Predicts Virus Adaptation**

Shu-Yang Jiang[1,2], Shi-Shun Zhao[1], Jun-Qing Wei[2,3], Sen Zhang[2], Zhongpeng Zhao[2], Yigang Tong[3], Wei Liu[2], Jianwei Wang[4,5*], Tao Jiang[2*], Jing Li[2*]

1. College of Mathematics, Jilin University, Changchun, Jilin 130012, China
2. State Key Laboratory of Pathogen and Biosecurity, Academy of Military Medical Science, Beijing 100071, China
3. Beijing Advanced Innovation Center for Soft Matter Science and Engineering (BAIC-SM), College of Life Science and Technology, Beijing University of Chemical Technology, Beijing 100029, China
4. NHC Key Laboratory of Systems Biology of Pathogens and Christophe Merieux Laboratory, National Institute of Pathogen Biology, Chinese Academy of Medical Sciences and Peking Union Medical College, Beijing 100730, China
5. Key Laboratory of Respiratory Disease Pathogenomics, Chinese Academy of Medical Sciences and Peking Union Medical College, Beijing 100730, China

*** Correspondence:**

Dr **Jianwei Wang**, Professor, Email: wangjw28@163.com,
Dr **Tao Jiang**, Professor, Email: jiangtao@bmi.ac.cn,
Dr **Jing Li**, Professor, Email: lj-pbs@163.com.

Supplementary methods, supplementary figures and supplementary tables

Contents

1 Supplementary methods......3
1.1 Performance evaluation of HMM tokenizer......3
1.2 Dimensionality reduction and clustering of embedded sequences......3
1.3 Statistical testing of significant differences......4
1.4 vBERT embedding-based immune escape analysis of IAV vaccine strains......4
1.5 vBERT embedding-based mutational effect analysis of single amino acid......5
1.6 Dataset retrieval in GIVAL......5
1.7 Prediction robustness evaluation of GIVAL for extreme sequence lengths......5
1.8 Validation and cross-species generalization test of GIVAL based on IAV from pandemics and IBVs......6
1.9 Protein structure prediction, alignment and visualization......6
1.10 Bayes inference of adaptation-important amino acid sites......6
1.11 Adaptation prediction of IAV HA RBD from mammalian hosts......7
1.12 Binding kinetics analysis of HA1-Glycan interactions by bio-layer interferometry (BLI)......8
1.13 Adaptation prediction and quantify of H5N1 mutations......8
1.14 Prediction of adaptation shift for monkeypox viruses......9
2 Supplementary figures and figure legends......10
3 Supplementary tables......30

1 Supplementary methods

1.1 Performance evaluation of HMM tokenizer

To evaluate the performance of HMM tokenizer, the HMM-tokenized vocabulary list of the sampled 100 000 sequences datasets and the whole 1.86 million sequences dataset was first obtained. The ratio of the vocabularies of whole-dataset list included in the sampled-dataset list was calculated for each family as the coverage without weight, and on this basis, using the frequency of each token in each family as weight, the coverage with weight was also calculated. The generated virtual dataset was also tokenized with HMM, and the starting, emit and transition probability was compared with the results of the real-sequences-based (real sequences represent the sequences in the HMM dataset) HMM tokenizer by calculating the cosine value of the angle between the vectors. Human H1, H3 and avian H5, H7, H9 IAV HA sequences were deduplicated with CD-HIT with a threshold of 99.5% and annotated with HA1 and HA2. The sequences were tokenized with HMM and embedded with vBERT to test the capacity of the HMM-based vBERT on capturing domain-function association by distinguishing tokens from hypervariable HA1 and conserved HA2 domain.

1.2 Dimensionality reduction and clustering of embedded sequences

Python package sklearn.manifold.TSNE and sklearn.decomposition.PCA (https://scikit-learn.org/stable/about.html#citing-scikit-learn) were utilized to perform t-distributed Stochastic Neighbor Embedding (t-SNE) or Principal Component Analysis (PCA) for dimensionality reduction of embeddings of the protein sequences. Two components extracted as t-SNE1 and t-SNE2 or PCA1 and PCA2 were normalized with the following formula (1):

$$X_{normalized} = (X - X_{min})/(X_{max} - X_{min}), (1)$$

On this basis, Python package sklearn.cluster.MiniBatchKMeans was utilized for MiniBatch K-Means clustering of the reduced two components and clustering indexes were calculated for evaluation. The clustering number can be defined or automatically selected with the following formula (2):

$$k_{best} = argmax_k[\frac{SSE_{k-1}-SSE_k}{SSE_k-SSE_{k+1}}], (2)$$

In equation (2), SSE_{k-1}, SSE_k and SSE_{k+1} represent the clustering SSE of $k-1$, k and $k+1$ clusters.

1.3 Statistical testing of significant differences

Python package scipy.stats.kruskal was utilized to perform statistical Kruskal-Wallis testing of the two sets of data. The difference showed in Figure 6D was tested with Python package scipy.stats.ttest_rel and scipy.stats.scipy.stats.wilcoxon, and other differences between two groups of data were tested with Kruskal-Wallis test. If P-value was less than 0.05, the two sets of data were considered to be significantly different.

1.4 vBERT embedding-based immune escape analysis of IAV vaccine strains

IAV vaccine reference strains of H1 or H3 serotype published by the WHO from 2021 to 2023 (https://www.who.int/publications/m/item/recommended-composition-of-influenza-virus-vaccines-for-use-in-the-2021-2022-northern-hemisphere-influenza-season, https://www.who.int/news/item/25-02-2022-recommendations-announced-for-influenza-vaccine-composition-for-the-2022-2023-northern-hemisphere-influenza-season and https://www.who.int/publications/m/item/recommended-composition-of-influenza-virus-vaccines-for-use-in-the-2023-2024-northern-hemisphere-influenza-season) were downloaded from NCBI and the circulating H1 and H3 strains from 2021 to 2023 were also extracted from the original dataset (including 2 079 sequences for H1N1 and 8 245 sequences for H3N2). Hemagglutinin (HA) sequences of the strains were reduced with t-SNE and clustered after embedded with vBERT. The clustering number was automatically selected. The ratio of H1 and H3 circulating strains in the same (defined as high homology with vaccine strains) or different (defined as low homology with vaccine strains) cluster with the reference strains were counted for each year respectively, to evaluate the immune escape of IAV in each year. The immune escape index was defined as the number of samples with low homology divided by the number of samples with high homology of each year.

1.5 vBERT embedding-based mutational effect analysis of single amino acid

Single-site-mutated sequences, preference or binding score and site entropy of IAV HA and SARS-CoV-2 Spike Receptor Binding Domain (RBD) were obtained from Deep Mutational Scanning (DMS) datasets (including 11 280 mutants for IAV HA and 3 994 mutants for SARS-CoV-2 Spike RBD). The sequences were embedded with vBERT and reduced with PCA to extract PCA1 of each sequence. For RBD sequences mutated with 20 amino acids at each important site, PCA1 of sequences with high and low binding score were compared and the two sets of PCA1 data were tested for significant differences. To evaluate the relationship between the site entropy and the variation of embeddings for HA sequences mutated with 20 amino acids at each site, the variation of the PCA1 values were calculated. On this basis, the variation of PCA1 at the sites with high and low site entropy were compared and tested for significant differences.

1.6 Dataset retrieval in GIVAL

The mapped dataset was retrieved based on the virus and protein name if the virus and protein name can be found in the sequence dataset. However, labels of some sequences were not complete and the dataset was retrieved based on the homology between the sequences if the mapped virus and gene cannot be found in the sequence dataset. The sequences that differ in length from the mapped reference sequence by no more than 10% were first screened. The AA pair vector of the screened sequences and the mapped reference sequence were respectively calculated. On this basis, PCA was utilized for dimensionality reduction of each vector and MiniBatch K-Means was utilized for clustering with clustering number automatically selected. The sequences in the same cluster with the mapped reference sequence were extracted and randomly sampled as the retrieved dataset.

1.7 Prediction robustness evaluation of GIVAL for extreme sequence lengths

To evaluate the prediction robustness for inputs with extreme sequence lengths, three distinct starting sites were selected on strain NC_007362.1, namely the first residue of the signal peptide, site 170 and 334 (excluding the signal peptide) respectively near the

RBD region and a relatively conserved region. For each of these starting sites, sequence fragments of varying lengths (30, 40, and 50 AAs) were extracted and predicted by GIVAL, with prediction performance subsequently assessed by calculating confusion matrices on the independent validation dataset.

1.8 Validation and cross-species generalization test of GIVAL based on IAV from pandemics and IBVs

IAV HAs from 2009 H1N1 pdm09 (collected and submitted from June 1st, 2009 to December 31st, 2009) and 1968 H3N2 (collected from January 1st, 1968 to December 31st, 1968) human pandemic and influenza B viruses (IBVs) from 2020 to 2023 were downloaded from GISAID. Data cleaning was performed to extract IAV HAs started from 'M' AA with more than 540 AAs and without unknown AAs and IBV HAs without unknown AAs. IAV and IBV HAs were deduplicated by CD-HIT respectively with thresholds of 99.5% and 99.0%. The segmented HA RBD and complete HA of strain NC_007362.1 were respectively inputted in GIVAL with IAV human H1, H3 and avian H5, H7 and H9 in the training dataset to establish the complete HA and segmented HA model. Considering the low homology between the IAVs and IBVs, complete IBV HAs were predicted with the complete HA model, and HA RBD segments were extracted from IAV from pandemics and predicted with the segmented HA model.

1.9 Protein structure prediction, alignment and visualization

ESM-fold was utilized for protein structure prediction and PyMol was utilized for structure visualization and alignment. The Root Mean Square Deviation (RMSD) value was calculated with cmd.align to compare the structure between different protein sequences. The structure of A/Texas/37/2024 (H5N1) HA (without the signal peptide) showed in Figure 5H was predicted with AlphaFold2, and other structures were predicted with ESM-fold.

1.10 Bayes inference of adaptation-important amino acid sites

Protein sequences were aligned and segmented into a list of single AAs for all AA sites. The conditional probability for every AA type at each site based on adaptation group

was calculated according to the following Bayesian formula with Laplace smoothing (3)-(4):

$$\lambda = 1, (3)$$

$$\mathrm{P}(X|Y) = \frac{count(X,Y)+\lambda}{count(Y)+\lambda N}, (4)$$

In equation (4), N represents the number of adaptation groups.

1.11 Adaptation prediction of IAV HA RBD from mammalian hosts

The HA from mammalian hosts other than humans (including 5 403 sequences) were mapped and the HA RBD segments were extracted from each sequence. The flexible label was predicted and the host-adaptive label of each sample was further analyzed based on the majority of adaptive hosts in the predicted flexible cluster. The ratio of human-adaptive labels was calculated for each serotype and each host. The result was visualized with a scatter plot and the larger radius of the point represents a larger number of samples. The human-adaptive risk index (HRI) was obtained for samples collected in each year period from each continent with the following formula (5)-(6):

$$HRI_{i,j_{\text{origin}}} = r_{i,j}(1 + \sqrt{log_{10}n_{i,j}}), (5)$$

$$HRI_{i,j} = \frac{HRI_{i,j_{\text{origin}}} - \min_{i,j}\left(HRI_{i,j_{\text{origin}}}\right)}{(\max_{i,j}\left(HRI_{i,j_{\text{origin}}}\right) - \min_{i,j}(HRI_{i,j_{\text{origin}}}))}, (6)$$

In equation (5)-(6), $HRI_{i,j}$, $r_{i,j}$ and $n_{i,j}$ respectively represent the human-adaptive risk index, ratio of human-adaptive labels and total sample number of year period i from continent j.

A/swine/Bakum/IDT1769/2003 and A/canine/Korea/01/2007 were selected as reference sequences of H3N2, and A/equine/Ohio/113461-1/2005 and A/canine/Florida/15592.1/2004 were selected as reference sequences of H3N8. A/Texas/55/2014/human/H3N2 was selected as reference sequence of H3 for human-adaptive IAVs. After predicting, the HA RBD structure of human- (A/canine/Florida/61156-2/2006) and avian-adaptive (A/canine/Maine/058124/2016) canine H3N8, the human- (A/equine/Santiago/TT9A/2018) and avian-adaptive (A/equine/China/Ulumuqi/2015) equine H3N8 were aligned for further analysis on

differential sites.

In order to analyze the different sites between the human- and avian-adaptive H3N2 and H3N8 samples, the dot product and importance value were calculated based on Bayes model for each amino acid site in HA RBD, respectively. Logo-plots of the top-30-important sites of human- and avian-adaptive samples were drawn using Python package logomaker.Logo to visualize the distribution of amino acids of each site.

1.12 Binding kinetics analysis of HA1-Glycan interactions by bio-layer interferometry (BLI)

The kinetics of the interaction were characterized by bio-layer interferometry (BLI) using a Gator™ Label-Free Bioanalysis System. For the assay, α2,3-linked (3′-SLNLN) and α2,6-linked (6′-SLNLN) sialylglycan receptors were loaded onto Streptavidin (SA) biosensors at 1.25 μg/mL. The ligand-coated biosensors were then dipped into a series of wells containing the HA1 protein analyte, prepared in a two-fold serial dilution from 1000 μg/mL to 15.625 μg/mL. All sensorgrams were corrected by subtracting data from a reference sensor to account for baseline drift. The processed data were globally analyzed using a 1:1 binding model fit. The equilibrium dissociation constant (KD) was subsequently calculated by the Gator Bio analysis software, and all curves were plotted for visualization using GraphPad Prism 10.0.

The recombinant HA1 protein of A/barn swallow/Hong Kong/D10-1161/2010 (H5N1) (40160-V08H1), A/equine/Gansu/7/2008 (H3N8) (40155-V08H1) and A/Texas/50/2012 (H3N2) (40354-V08H1) were purchased from Sino Biological. The biotinylated glycan ligands (3′-SLNLN and 6′-SLNLN) were kindly provided by Researcher Yuhai Bi from the Institute of Microbiology, Chinese Academy of Sciences.

1.13 Adaptation prediction and quantify of H5N1 mutations

Based on the sampled human H1, H3 (750 sequences each) and avian H5, H7 and H9 (500 sequences each) HA RBD sequences, Bayes analysis was first conducted to obtain the site importance and amino acid distribution in human- and avian-adaptation sequences. According to the human-adaptive amino acid distribution of the selected

sites, 30 000 mutations were first generated based on the reference strain (A/Texas/37/2024(H5N1)). The adaptation of the generated mutations was predicted and quantified with GIVAL. Based on the mutations with avian adaptation score (AAS) lower than the given threshold value, ablation methods were conducted to mutate one single site back to the wild-type amino acid each time and the site effect index were calculated with the following formula (7)-(8):

$$site\ effect\ index_{origin} = \frac{\sum_{i=1}^{N}(AAS_{new} - AAS_{origin})}{N}, (7)$$

$$site\ effect\ index = \frac{site\ effect\ index_{origin} - min\ (site\ effect\ index_{origin})}{max(site\ effect\ index_{origin}) - min\ (site\ effect\ index_{origin})}, (8)$$

In equation (7)-(8), N represents the number of mutations with low avian adaptation score, AAS_{new} represents the avian adaptation score of the regenerated mutation and AAS_{origin} represents the avian adaptation score of the firstly generated mutation.

On this basis, less sites with high effect index were selected from the 30 sites and for each mutation, the remaining sites were mutated back to the wild-type amino acids. The adaptation risk of the regenerated mutations was evaluated with GIVAL and the high-risk mutations were listed and further analyzed for the key amino acids and their sites. To further analyze the relationship between the high-risk mutations and the circulating strains, the H5N1 IAV HAs circulated in 2025 were downloaded from GISAID and data cleaning was performed to extract sequences without unknown AAs. The sequences with the high-risk mutations were selected and analyzed.

1.14 Prediction of adaptation shift for monkeypox viruses

Ten proteins of monkeypox viruses were selected based on the current study and score calculated with 48-dimensional dinucleotide (DNT) vector and vBERT embeddings. The score was calculated with the following formula (9)-(10):

$$Score_{DNT} = \sqrt{\frac{1}{48}\sum_{i=1}^{48} Var(DNT_i)} + \frac{1}{50} ED_{clade\ I,clade\ II}, (9)$$

$$Score_{vBERT} = Var(PCA1) + \frac{1}{5} ED'_{clade\ I,clade\ II}, (10)$$

In equation (9)-(10), $ED_{clade\ I,clade\ II}$ and $ED'_{clade\ I,clade\ II}$ are the Euclidean Distance of the center of 48-dimensional DNT vector and 2-dimensional PCA vector of

vBERT embedding between clade I and clade II samples.

The adaptation score and degree of the ten proteins were predicted based on GIVAL with samples from clade I before 2022 and clade II before 2024 as training set (including 300 sequences for each protein) and clade I after 2022 (including 2022) and clade II in 2024 as validation set (including 318 sequences for each protein). The parsed protein sequences downloaded from NCBI (including 2 150 sequences for each protein) were predicted with the trained models. Type I and II (type I represents higher pathogenicity and lower transmissibility, and type II represents the lower pathogenicity and higher transmissibility) adaptation score of each sample was calculated with the vector of FC.

Adaptation degree was calculated with the ratio of type-II-adaptative protein in the 10 proteins. On this basis, the average adaptation score and degree of each country, continent, year and clade were obtained.

To further study the adaptation shift, the vBERT embeddings of sampled monkeypox dataset were extracted and reduced to PCA1 and PCA2. The samples were labeled with clade I before 2022, clade I after (and including) 2022, clade II before 2022 and clade II after (and including) 2022. The cosine value between vector from clade Ia (strain hMpxV/DRC/OHSU-01-P5/1978|EPI_ISL_13058456|1978) to Ib (strain hMpxV/Kenya/KEMRI-00107/2024|EPI_ISL_19345034|2024-07-25) and vector from clade IIa (strain hMpxV/cynomolgus_monkey/USA/un-WRAIR7-61-P2/1962|EPI_ISL_13056556|1962) to IIb B.1.20 (strain hMpxV/Australia/VIC-VIDRL-9428/2024|EPI_ISL_19459746|2024-05-14) were obtained to study the similarity between the adaptation shift of clade I and II.

2 Supplementary figures and figure legends

Supplementary Figure 1. Pipeline of establishment and evaluation of vBERT for virus protein sequences embedding.

The workflow of the establishment and evaluation of vBERT can be divided into five

parts. **A,** Virus dataset parsing was conducted for sequence deduplication, labeling and knowledge-based sampling of viruses from different families. **B,** Sequence tokenization and segmentation were conducted with HMM tokenizer for sequence verbalization and creation of vocabulary list and pretraining dataset. **C,** vBERT model was pretrained based on the vocabulary list and pretraining dataset. **D,** Optimized parameters were selected based on models pretrained with different datasets, sequence parsing methods, pretraining parameters and model frameworks. **E,** Embeddings of the test dataset extracted from different models were evaluated and compared based on the clustering indexes.

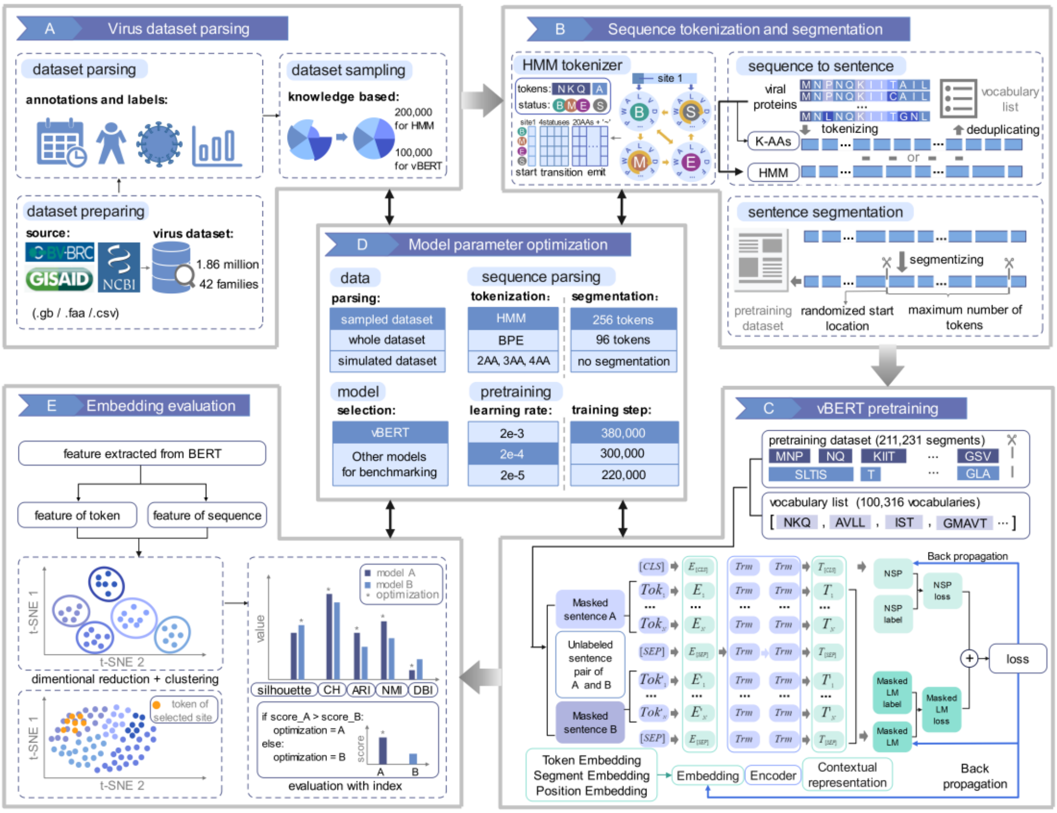

Supplementary Figure 2. Spatiotemporal distribution of the dataset.

The distribution of collection year and continent of different types of viruses was visualized with sankey plot.

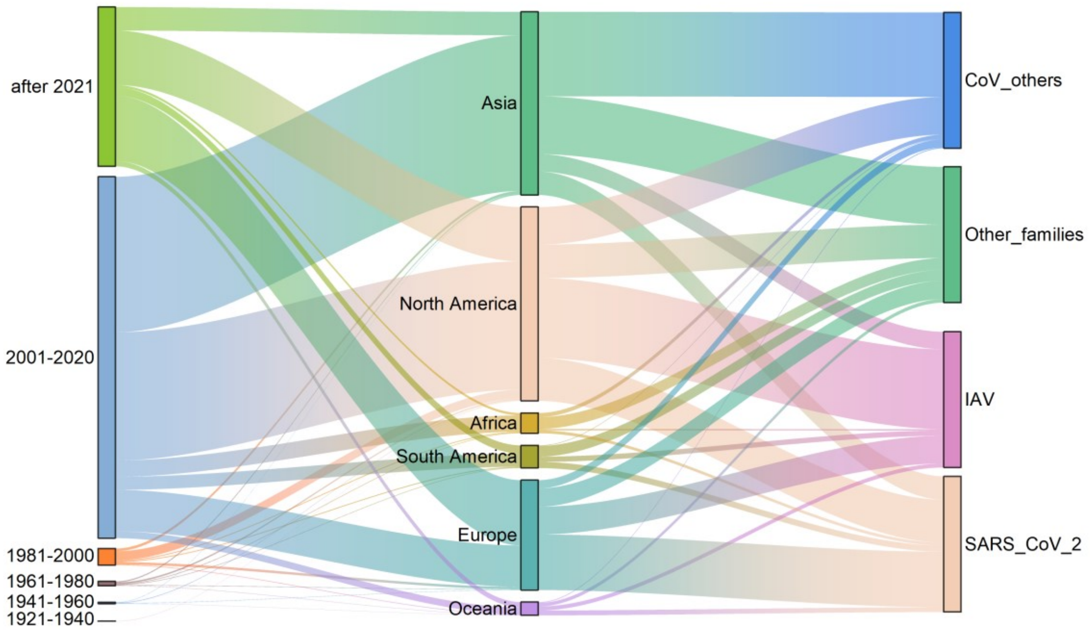

Supplementary Figure 3. Distribution of dataset before and after sampling.

The number of sequences from each family was respectively counted for the whole dataset (after deduplicated) **(A)** and the sampled HMM dataset **(B)**. The number of sequences in each family in sampled HMM dataset (sampled dataset) and randomly sampled dataset (including same number of sequences with sampled HMM dataset) were compared with five indexes, respectively Range (R), skewness (SKEW), kurtosis (KURT), Coefficient of Variation (CV) and Standard Deviation (SD) based on sequences in all, 50% and 25% of the families in the two datasets **(C)**. The Simpson Index of the vBERT pretraining dataset, HMM training dataset and the whole dataset was calculated based on frequence of each viral family **(D)**, and the Simpson Index and the normalized Simpson Index of the three datasets were calculated based on the sample number in each CD-HIT cluster (with threshold of 90%) for each viral family **(E)**.

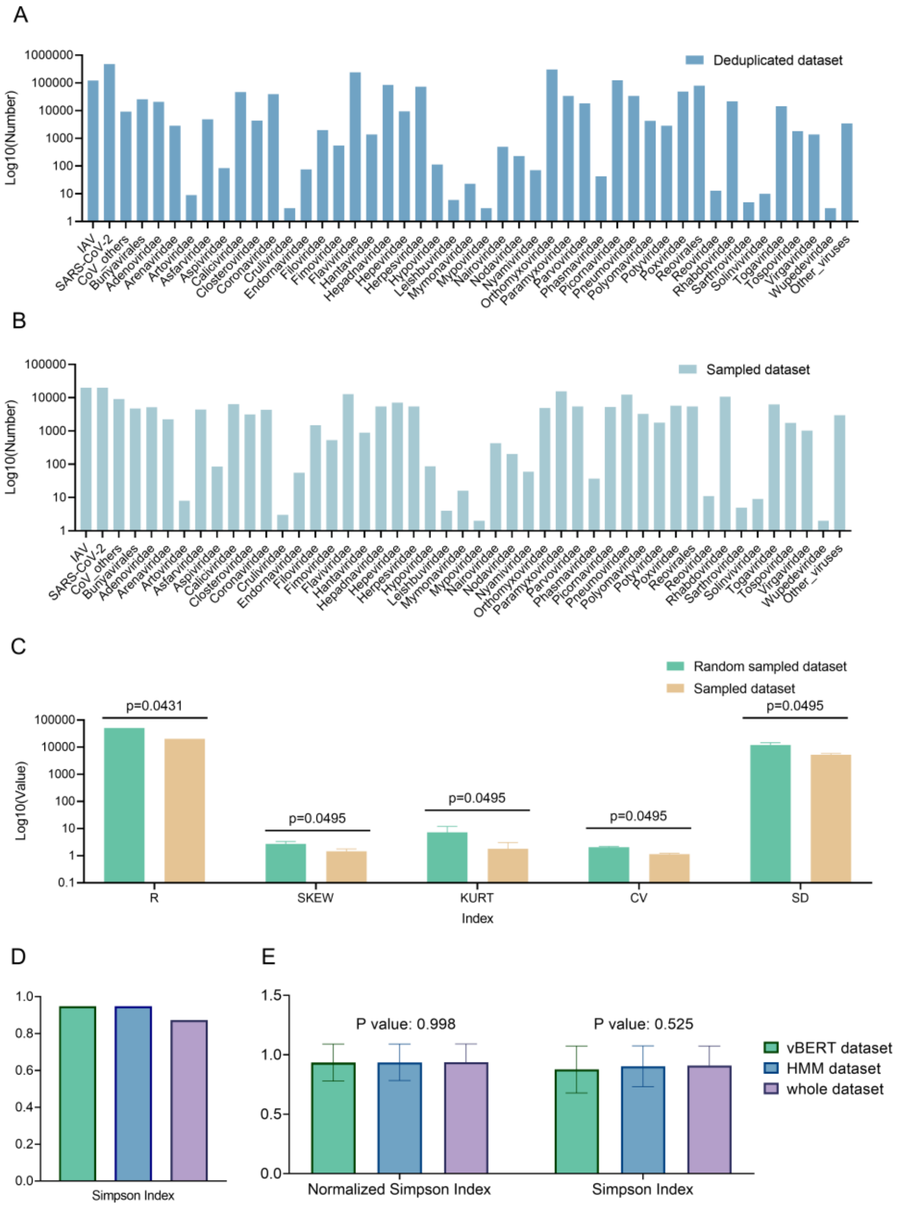

Supplementary Figure 4. Performance of HMM tokenizer on stability.

The coverage without weight of the HMM vocabulary list for each type of virus sequences in the whole dataset was counted, respectively **(A)**. The distribution of frequency of top 50 vocabularies in each family of RNA **(B)** and DNA **(C)** viruses were visualized with ridge plot.

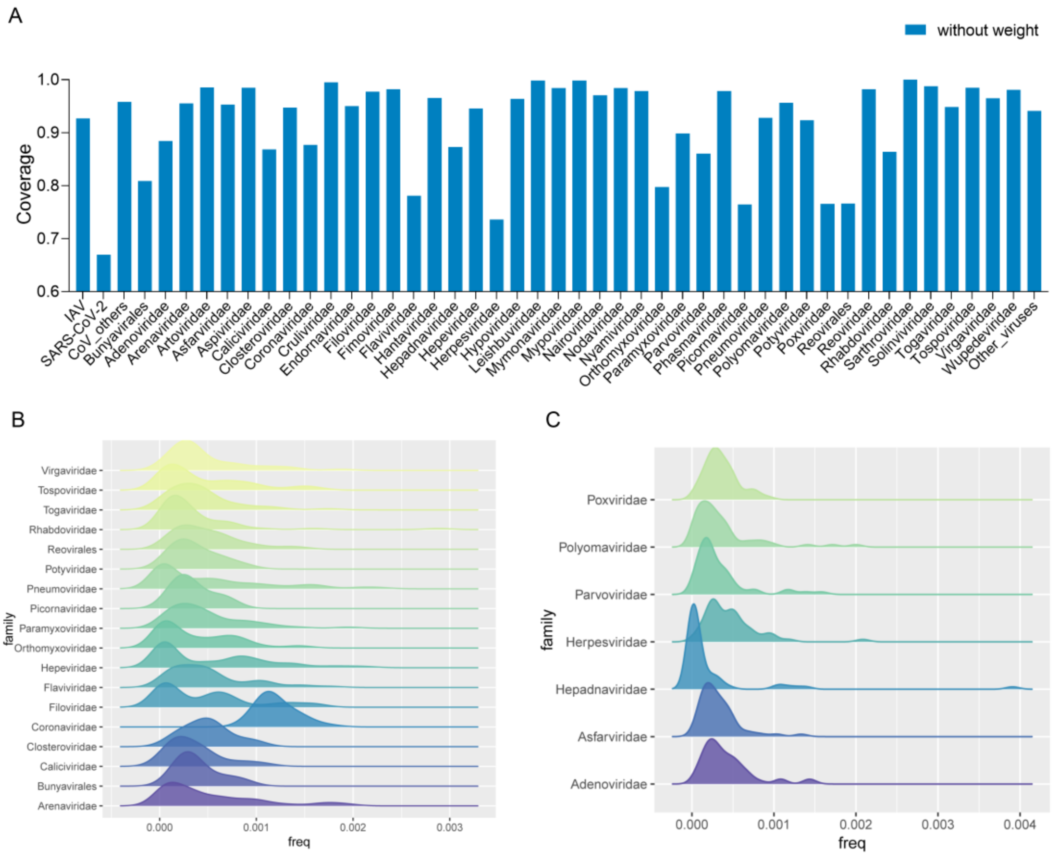

Supplementary Figure 5. Embedding performance of models other than vBERT-optimized on IAV HA.

Reduced two components with t-SNE from the vBERT-220,000-step **(A)**, vBERT-300,000-step **(B)**, vBERT-96-tokens **(C)**, vBERT-no-segmentation **(D)**, vBERT-lr2e-3 **(E)**, vBERT-lr2e-5 **(F)**, vBERT-simulated-dataset **(G)**, vBERT-whole-dataset **(H)**, vBERT-2AA **(I)**, vBERT-3AA **(J)**, vBERT-4AA **(K)** and vBERT-BERT-tiny **(L)**, vBERT-BERT-medium **(M)**, Transformer **(N)**, ESM-2 **(O)**, proteinBERT **(P)**, DNABERT-2 **(Q)**, vBERT-BPE **(R)** embedded IAV HA of sequences from different serotypes were visualized respectively.

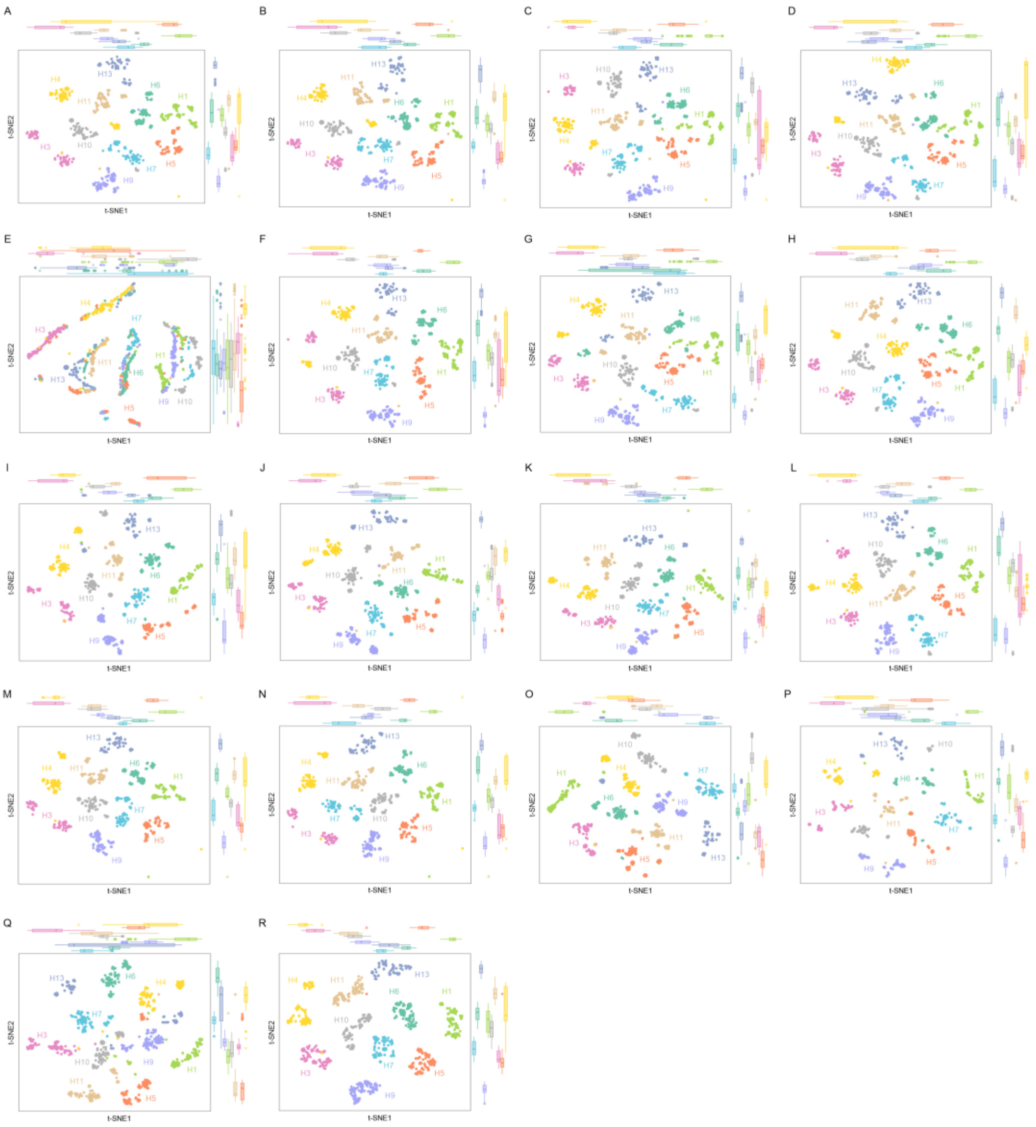

Supplementary Figure 6. Embedding performance of models other than vBERT-optimized on SARS-CoV-2 Spike RBD.

Reduced two components with t-SNE from the vBERT-220,000-step **(A)**, vBERT-300,000-step **(B)**, vBERT-96-tokens **(C)**, vBERT-no-segmentation **(D)**, vBERT-lr2e-3 **(E)**, vBERT-lr2e-5 **(F)**, vBERT-simulated-dataset **(G)**, vBERT-whole-dataset **(H)**, vBERT-2AA **(I)**, vBERT-3AA **(J)**, vBERT-4AA **(K)** and vBERT-BERT-tiny **(L)**, vBERT-BERT-medium **(M)**, Transformer **(N)**, ESM-2 **(O)**, proteinBERT **(P)**, DNABERT-2 **(Q)**, vBERT-BPE **(R)** embedded SARS-CoV-2 Spike RBD of sequences from different types were visualized respectively.

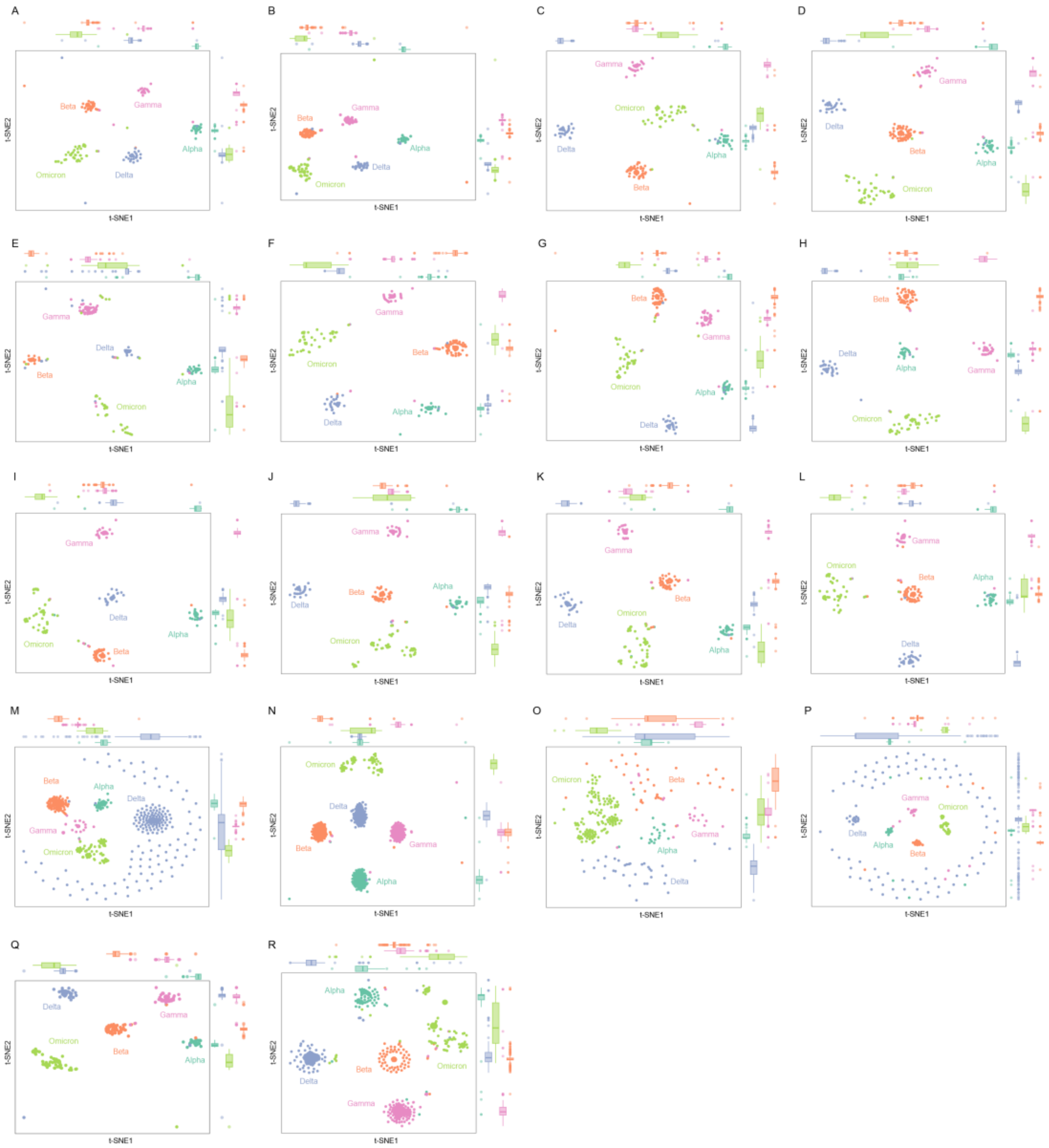

Supplementary Figure 7. Token embedding performance of vBERT and Word2Vec at important sites in IAV nucleoprotein (NP).

Two components of tokens of site 16, 313, 319 and 357 of IAV NP were reduced by PCA **(A)** and distribution of the tokens of site 16 **(B)**, 319 **(C)** and 357 **(D)** and tokens from all clusters of IAV NP were also compared by visualizing the reduced two components of t-SNE based on embedding extracted from vBERT, and the maximum ratio of tokens at the important sites in the same cluster was calculate. Same visualization was also conducted based on embedding extracted from Word2Vec **(E-H)** and vBERT-BPE **(I-L)**.

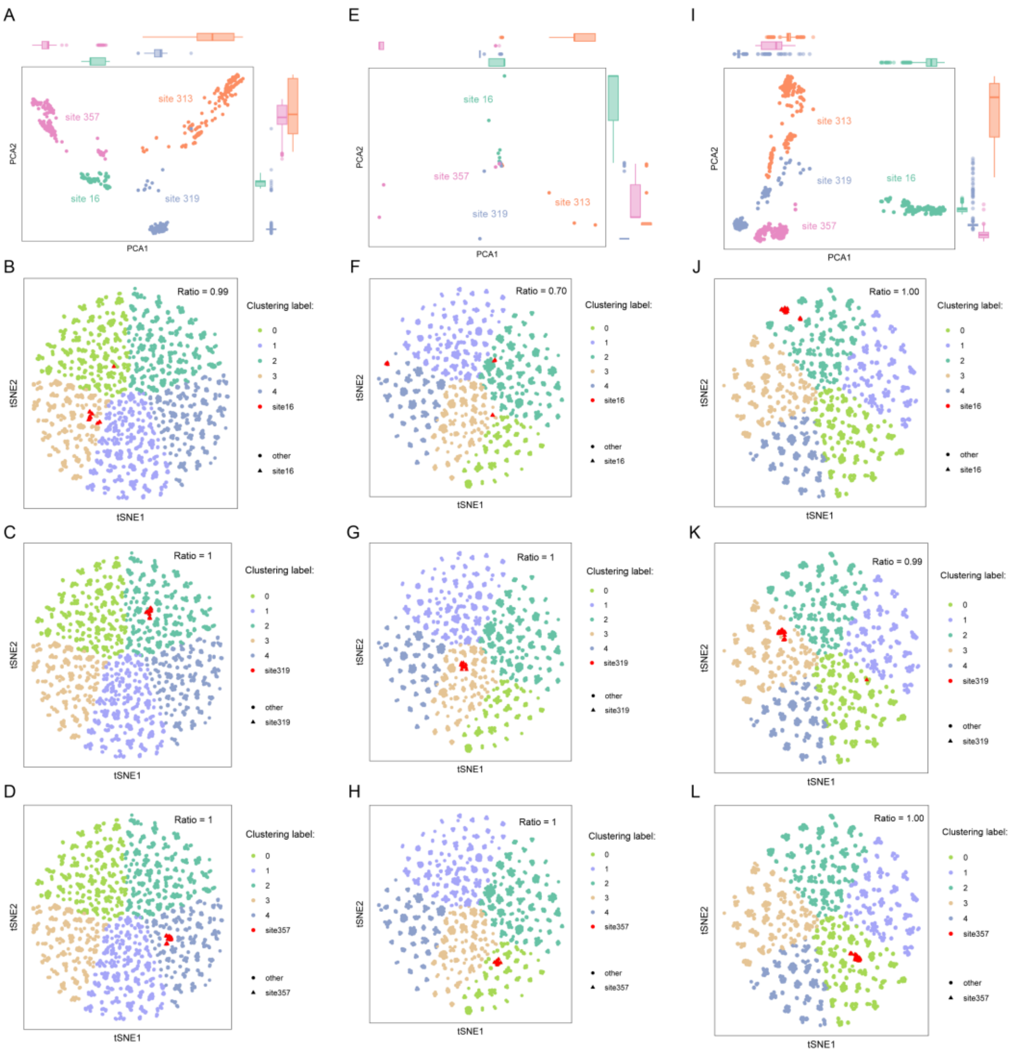

Supplementary Figure 8. Distribution of vBERT-embedded HMM tokens on IAV HA1 and HA2.

The occurrence counts of the top four most frequently appearing tokens on HA1 and HA2 of H1N1 **(A)**, H3N2 **(B)**, H5N1**(C)**, H7N9 **(D)** and H9N2 **(E)** were calculated. The occurrence count is the minimum of the occurrences in HA1 and HA2 for each token. Reduced two components with PCA of vBERT-embedded token with the highest **(F-J)** and the second highest **(K-O)** occurrence counts on HA1 and HA2 of the five serotypes were visualized.

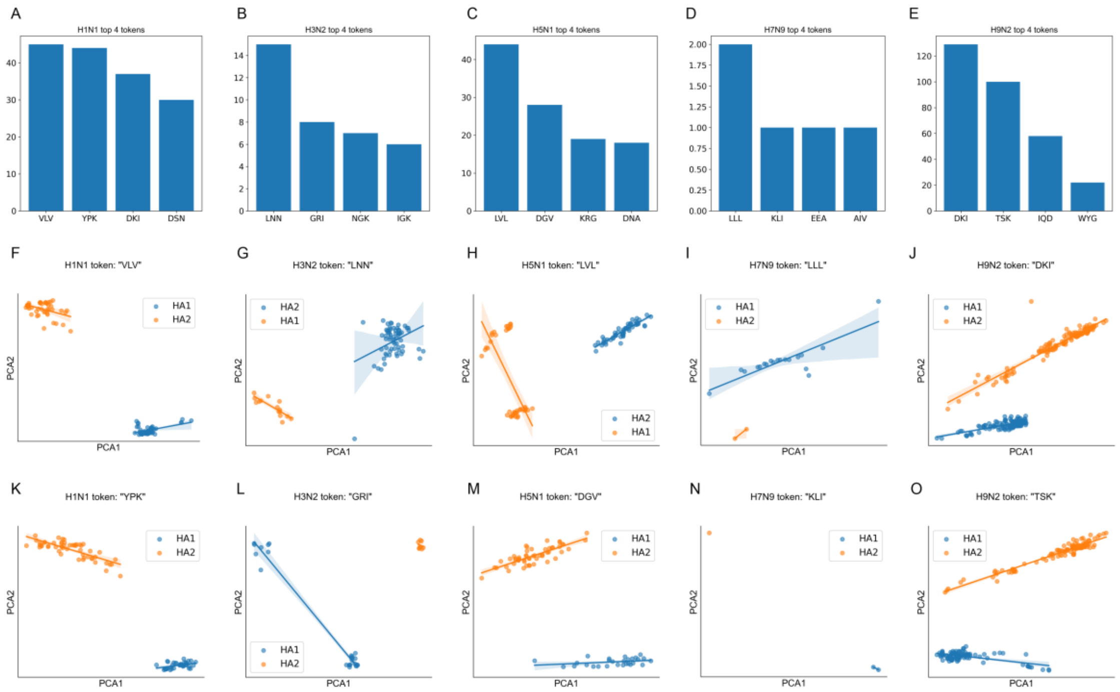

Supplementary Figure 9. Evaluation of immune escape and receptor binding ability of IAV HA and SARS-CoV-2 Spike based on vBERT embedding.

Reduced two components with t-SNE of vBERT embedding of HA protein from WHO influenza vaccine reference sequences and circulating influenza sequences of serotype H1 **(A)** and H3 **(B)** from 2021 to 2023 were visualized. Reduced one component with PCA of vBERT embedding of sequences from SARS-CoV-2 Spike Deep Mutational Scanning dataset with site 339, 449, 452 and 505 mutated singly with 20 amino acids was obtained, and PCA1 of sequences with high and low binding score were compared for each site **(C)**.

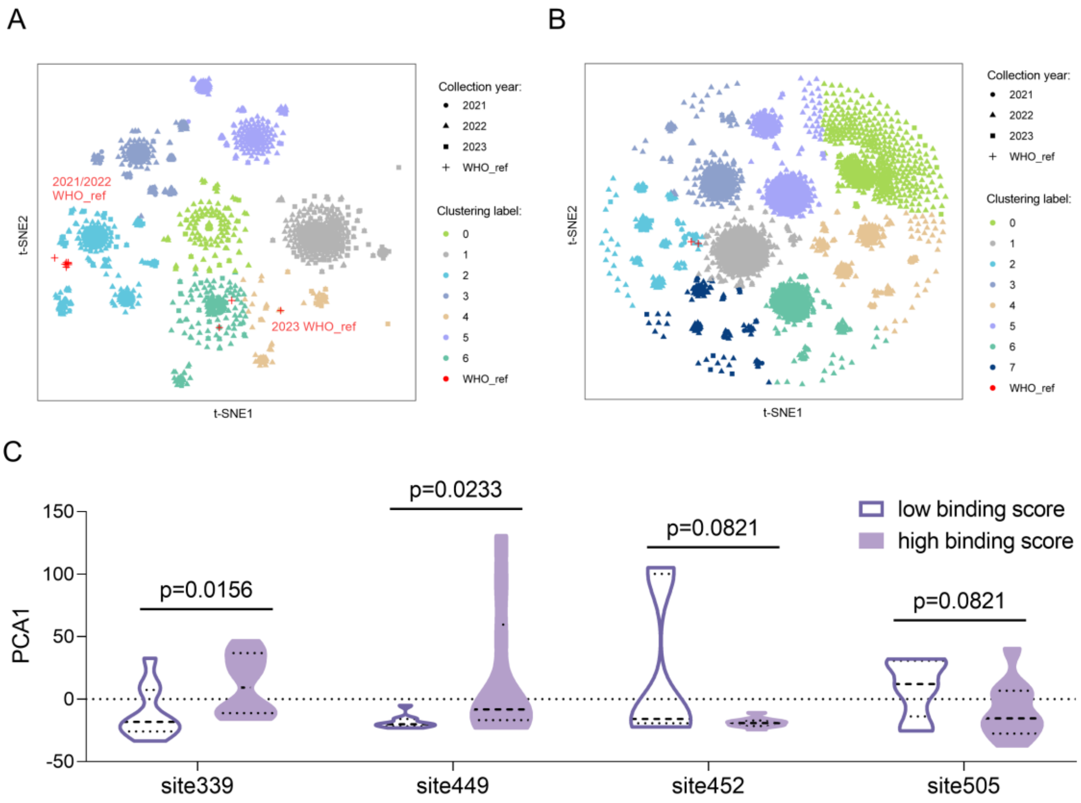

Supplementary Figure 10. Distribution of flexible labels of SARS-CoV-2 Spike RBD sequences in GIVAL.

Two components of embedding of Spike RBD whole **(A)** and sampled **(B)** dataset from different flexible labels were reduced with PCA. The Artiodactyla (ART) -, Chiroptera (CHI) -, Suiformes (SUI) - and Primates (PRI) -adaptive ratio of each type of sampled sequences from each flexible label was calculated and visualized with scatter pie plot **(C)**. Reduced two components with PCA of the vBERT-embedded Spike RBD of PDF-2180, NeoCoV, MERS-CoV, SARS-related and SARS-CoV-2 were visualized **(D).** Phylogenetic analysis was conducted for Spike RBD of PDF-2180, NeoCoV, MERS-CoV and SARS-related samples **(E)**.

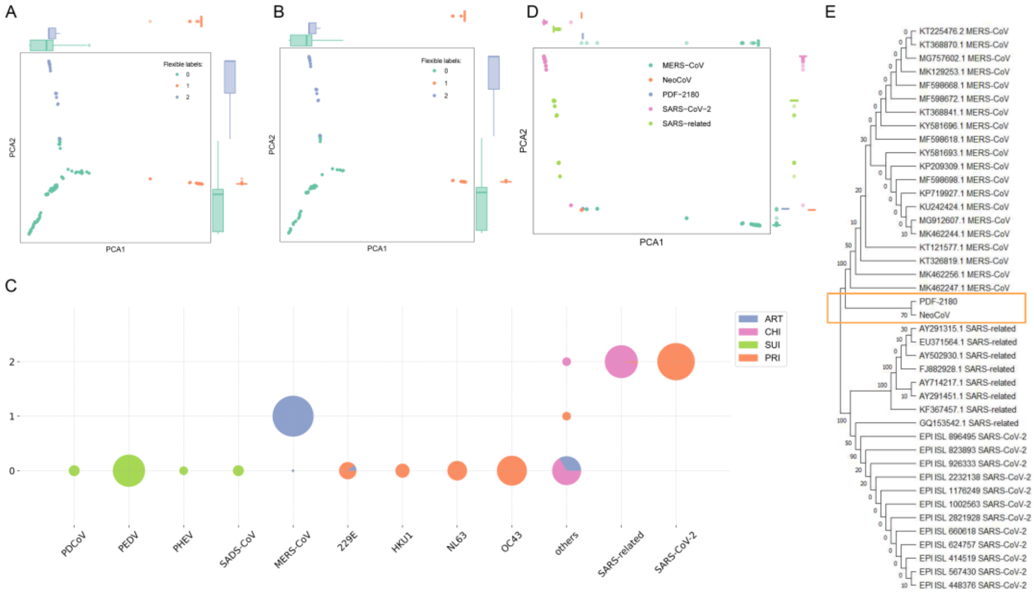

Supplementary Figure 11. Distribution of flexible labels of IAV HA RBD sequences in GIVAL.

Two components of embedding of HA RBD sequences from different flexible labels were reduced with PCA **(A)**. The human- and avian-adaptive ratio of each type of viruses from each flexible label was calculated and visualized with scatter pie plot **(B)**. Same visualization was conducted based on sampled HA RBD sequences **(C and D)**.

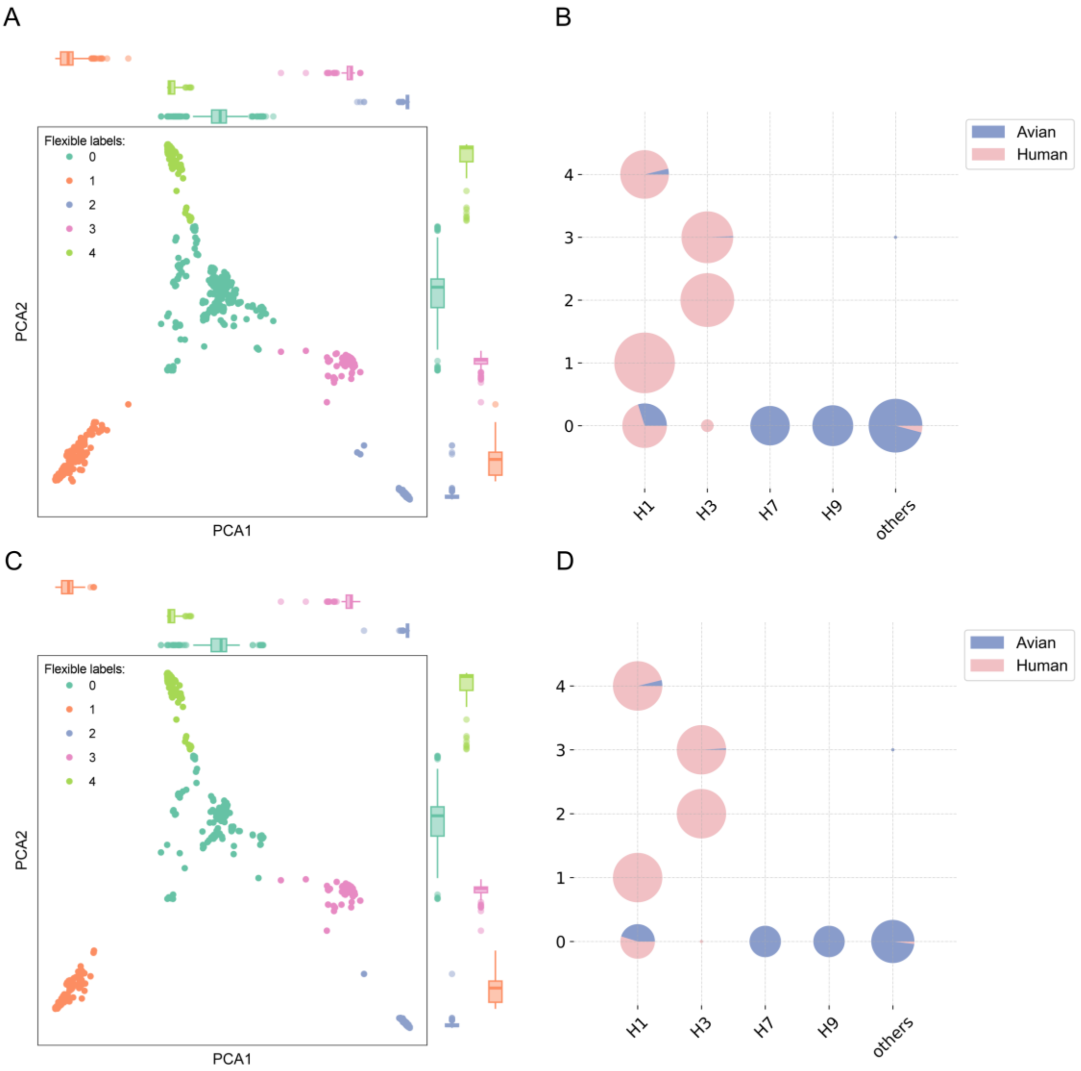

Supplementary Figure 12. Label fault tolerance of ResNet predictor based on flexible and specified labels of IAV HA.

The confusion matrices on the independent validation set of models based on specified host labels with error rate of label of training human H3 of 0 **(A)**, 0.05 **(B)**, 0.10 **(C)**, 0.15 **(D)** and 0.20 **(E)** were calculated and the confusion matrix based on flexible labels with the above error rates of labels was also calculated **(F)**.

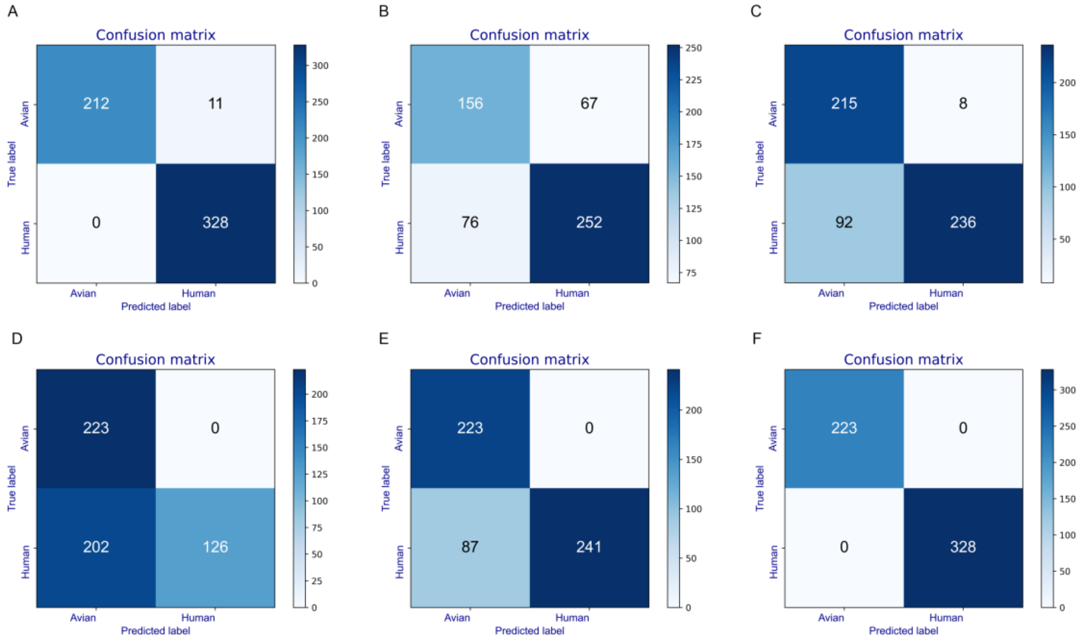

Supplementary Figure 13. Performance of ResNet predictor of IAV HA RBD on three validation sets.

The confusion matrix **(A-C)** and ROC with AUC **(D)** of the three cross validation sets based on the HA RBD model were obtained. The number of human H1, human H3, human-adaptive samples other than H1 and H3 serotype and avian-adaptive in each flexible cluster before **(E)** and after sampling **(F)** were counted based on the clustering results of HA RBD.

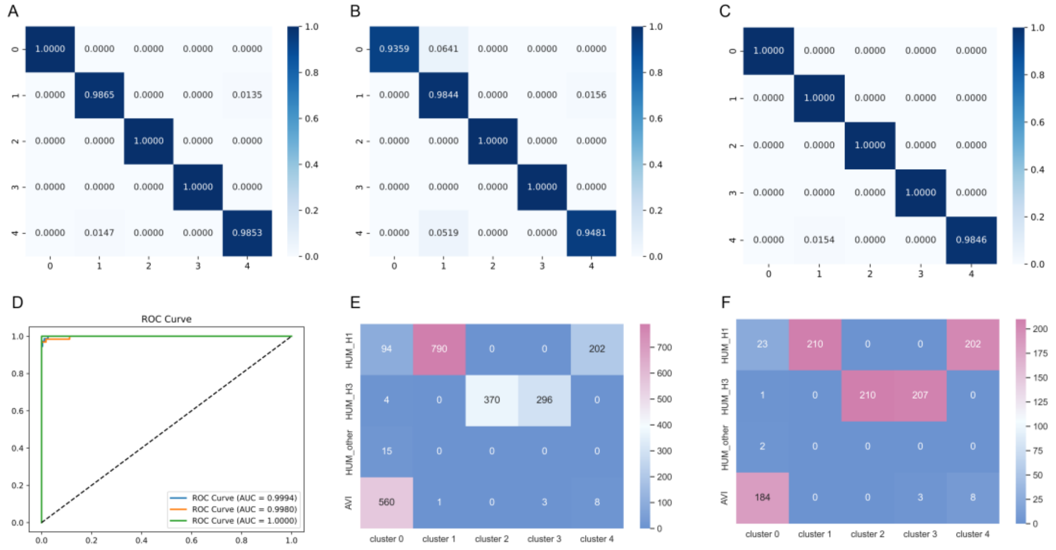

Supplementary Figure 14. Performance of ResNet predictor of IAV HA complete

sequence on three validation sets.

The confusion matrix **(A-C)** and ROC with AUC **(D)** of the three cross validation sets based on the HA model were obtained. The number of human H1, human H3, human-adaptive samples other than H1 and H3 serotype and avian-adaptive in each flexible cluster before **(E)** and after sampling **(F)** were counted based on the clustering results of HA.

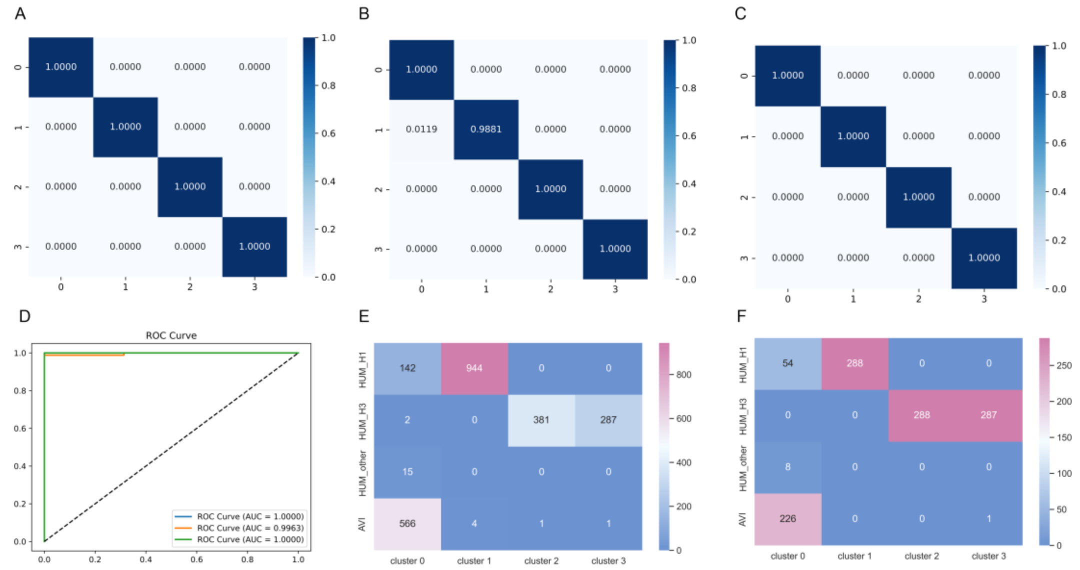

Supplementary Figure 15. Performance of ResNet predictor of SARS-CoV-2 Spike RBD on three validation sets.

The confusion matrix **(A-C)** and ROC with AUC **(D)** of the three cross validation sets based on the Spike RBD model were obtained. The number of ART-, CHI-, SUI-, PRI-adaptive and SARS-CoV-2 samples in each flexible cluster before **(E)** and after sampling **(F)** were counted based on the clustering results of Spike RBD.

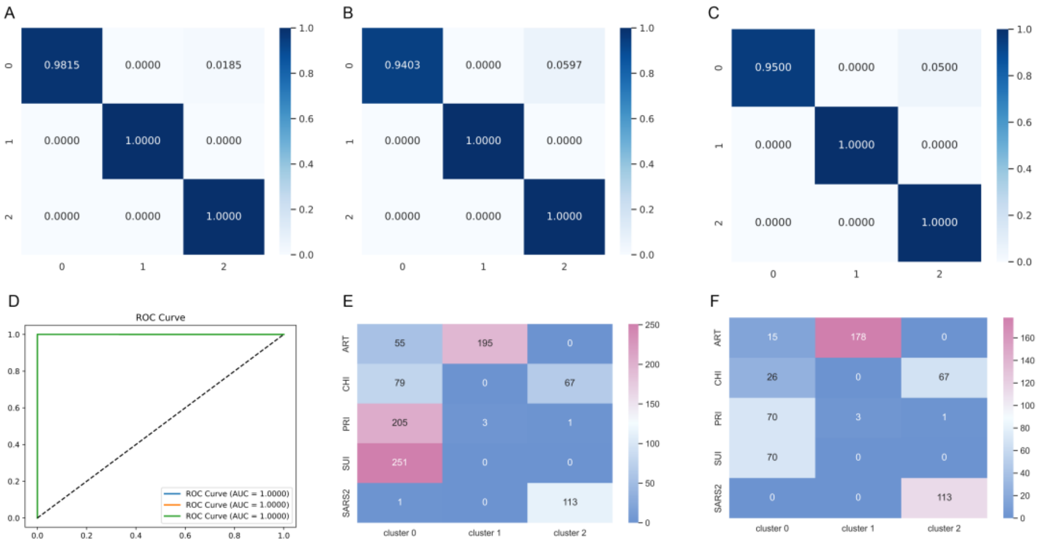

Supplementary Figure 16. Prediction performance of GIVAL on input with extreme sequence lengths.

The confusion matrices of GIVAL on the independent validation dataset based on input sequence from the first AA site of the signal peptide of strain NC_007362.1 with lengths of 30 **(A)**, 40 **(B)** and 50 **(C)** AAs were calculated. The confusion matrices based on the input from site 170 **(D-F)** and 334 **(G-I)** (excluding the signal peptide) with the above lengths were also calculated.

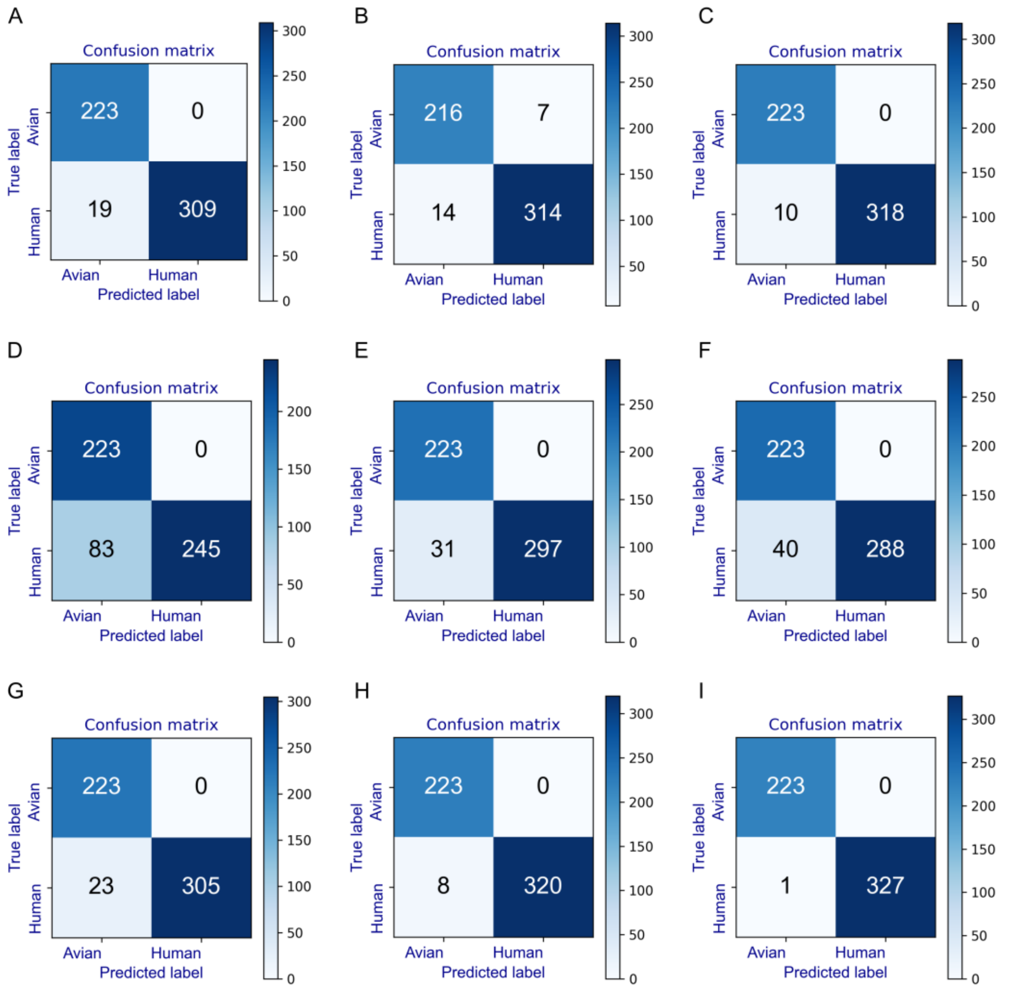

Supplementary Figure 17. Prediction of IAVs from pandemics and IBVs.

The prediction pipeline **(A)** and results **(B)** of 2009 H1N1 and 1968 H3N2 IAV HA RBD segments and IBV HA complete sequences based on GIVAL were visualized.

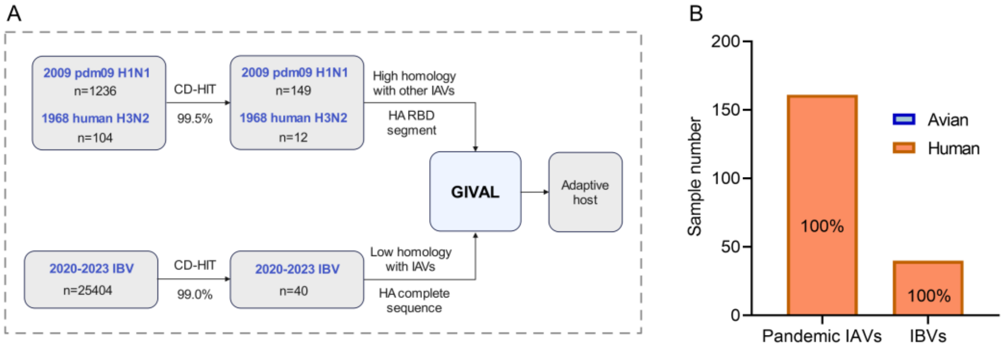

Supplementary Figure 18. Key sites analysis and structure prediction of

mammalian H3N2, H3N8 and avian H5N1 mutations.

The distribution of amino acids of top-30-important sites of human- and avian-adaptive H3N2 **(A)** and H3N8 **(B)** predicted by GIVAL were calculated. The RMSD between the HA RBD structure of H3N2 reference sequences and other human-adaptive swine H3N2 and avian-adaptive canine H3N2 **(C)** was visualized. The HA RBD structure of human- (pink) and avian-adaptive (blue) canine **(D)** and equine **(E)** H3N8 were aligned respectively and the site with different amino acids distribution were emphasized with sticks.

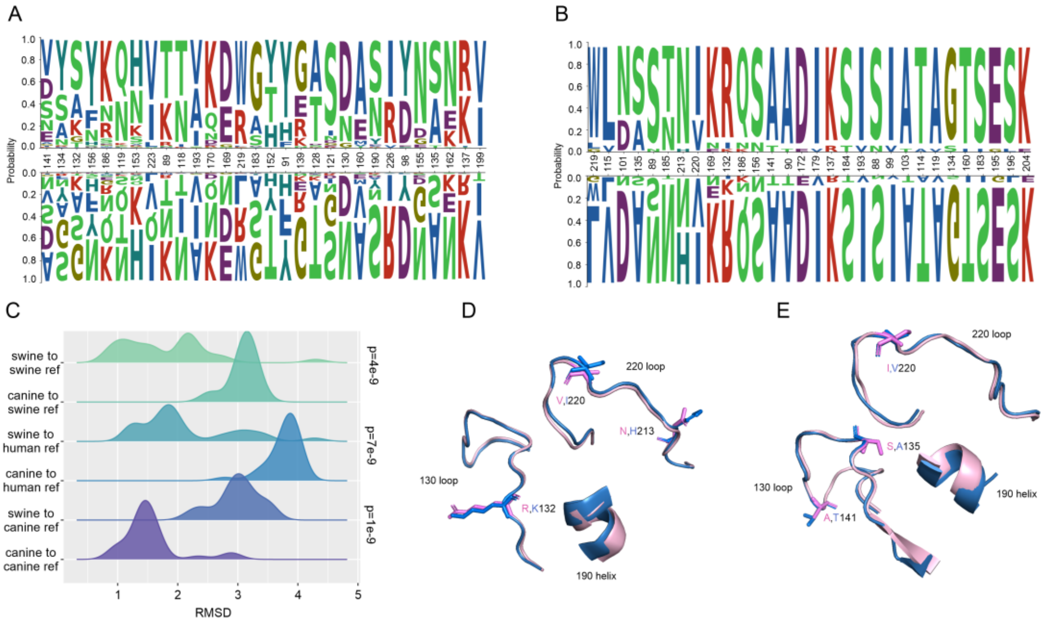

Supplementary Figure 19. Receptor-binding properties of IAV HA1 proteins to biotinylated glycan ligands (3′-SLNLN and 6′-SLNLN)

Bio-layer interferometry (BLI) assays were performed to analyze interactions between the HA1 protein of avian H5N1 IAV and biotinylated glycans 3′-SLNLN **(A)** and 6′-SLNLN **(B)**. Similar binding assays were conducted for the HA1 proteins of human H3N2 **(C, D)** and equine H3N8 IAVs **(E, F)** with the respective glycans.

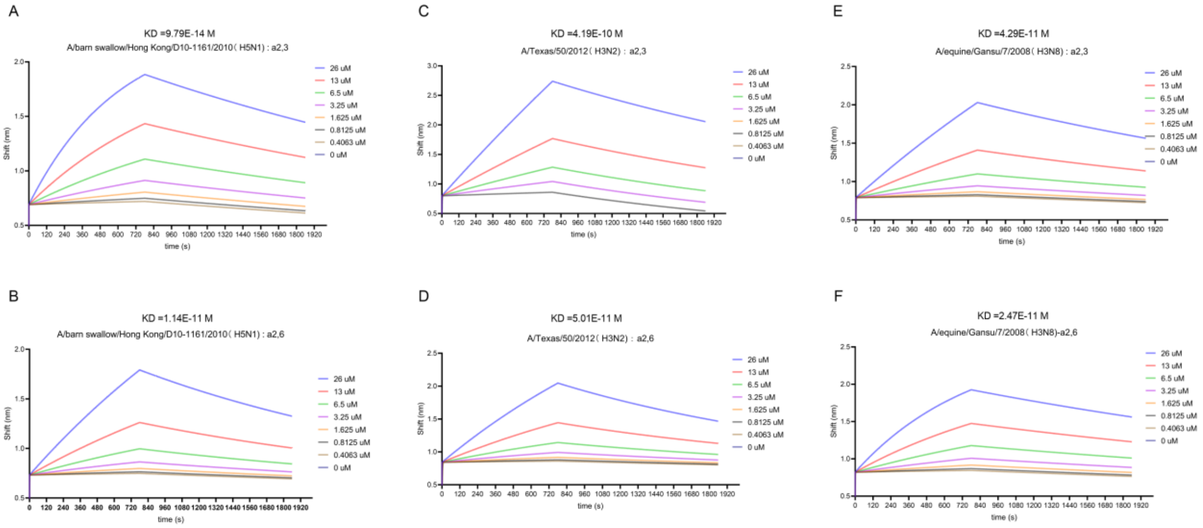

Supplementary Figure 20. Score of top-30 proteins of monkeypox viruses

Top-30 important proteins of monkeypox viruses were selected based on score of vBERT embedding **(A)** and DNT **(B)**.

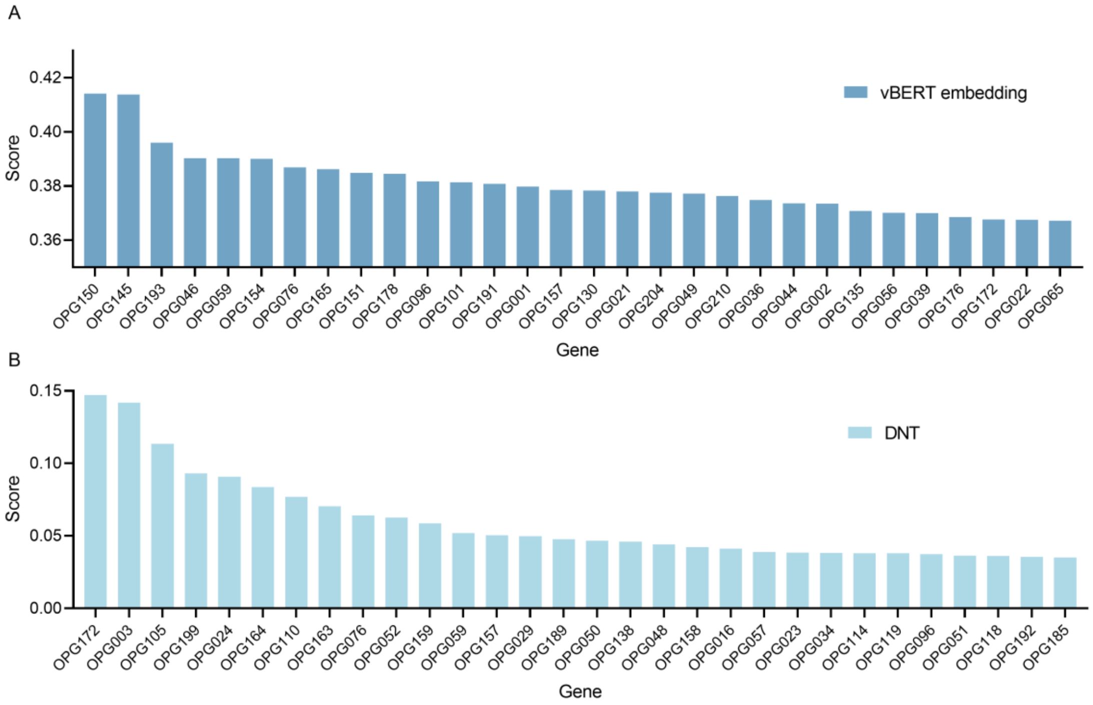

Supplementary Figure 21. Confusion matrices of validation set of 10 proteins of monkeypox virus samples.

Confusion matrices of validation set of monkeypox virus samples based on OPG002 **(A)**, OPG015 **(B)**, OPG019 **(C)**, OPG031 **(D)**, OPG034 **(E)**, OPG049 **(F)**, OPG100 **(G)**, OPG130 **(H)**, OPG170 **(I)** and OPG172 **(J)** sequences were respectively obtained (0

represents type I and 1 represents type II adaptation).

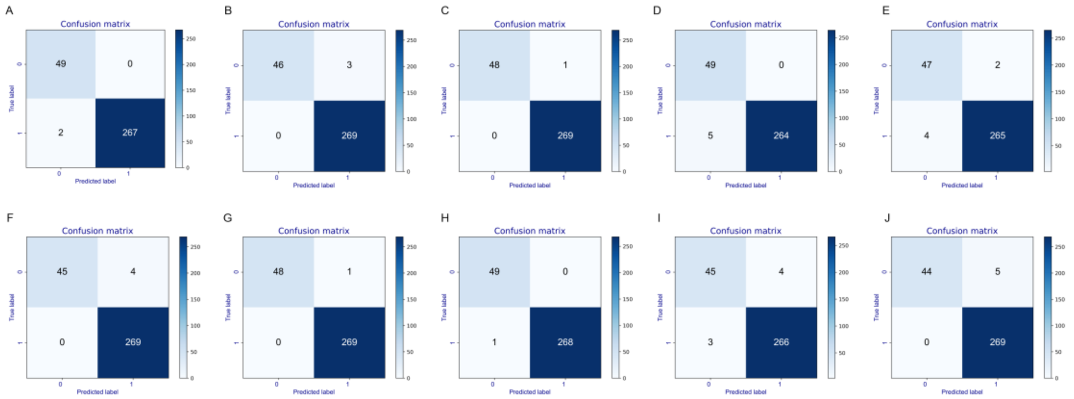

Supplementary Figure 22. Distribution of reduced Fully Connected layer (FC) vector of 10 proteins of monkeypox virus samples.

Reduced three components with PCA from FC vector of type I and II monkeypox virus samples in training set and clade Ia and Ib samples in validation set based on OPG002 **(A)**, OPG015 **(B)**, OPG019 **(C)**, OPG031 **(D)**, OPG034 **(E)**, OPG049 **(F)**, OPG100 **(G)**, OPG130 **(H)**, OPG170 **(I)** and OPG172 **(J)** sequences were respectively obtained.

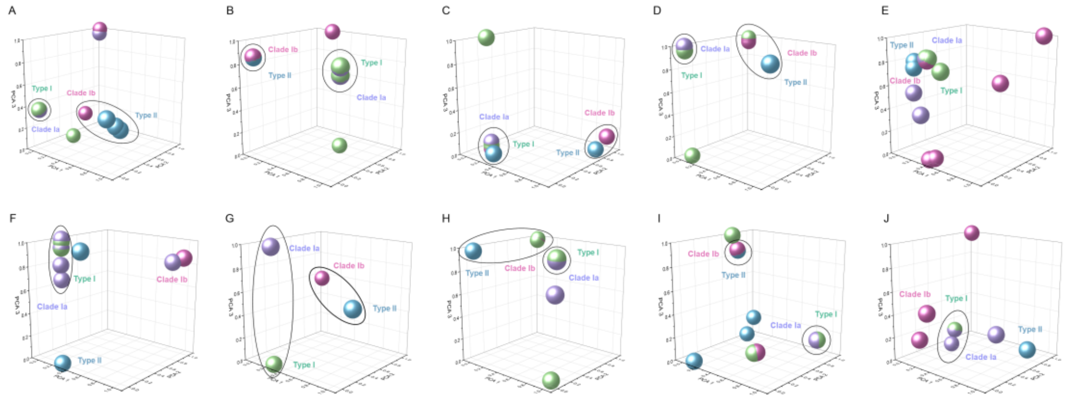

Supplementary Figure 23. Spatiotemporal distribution of each clade of monkeypox virus dataset.

The distribution of collection year and continent of clade Ia, Ib, IIa, IIb A, IIb B, IIb C in monkeypox virus dataset downloaded from GISAID were obtained.

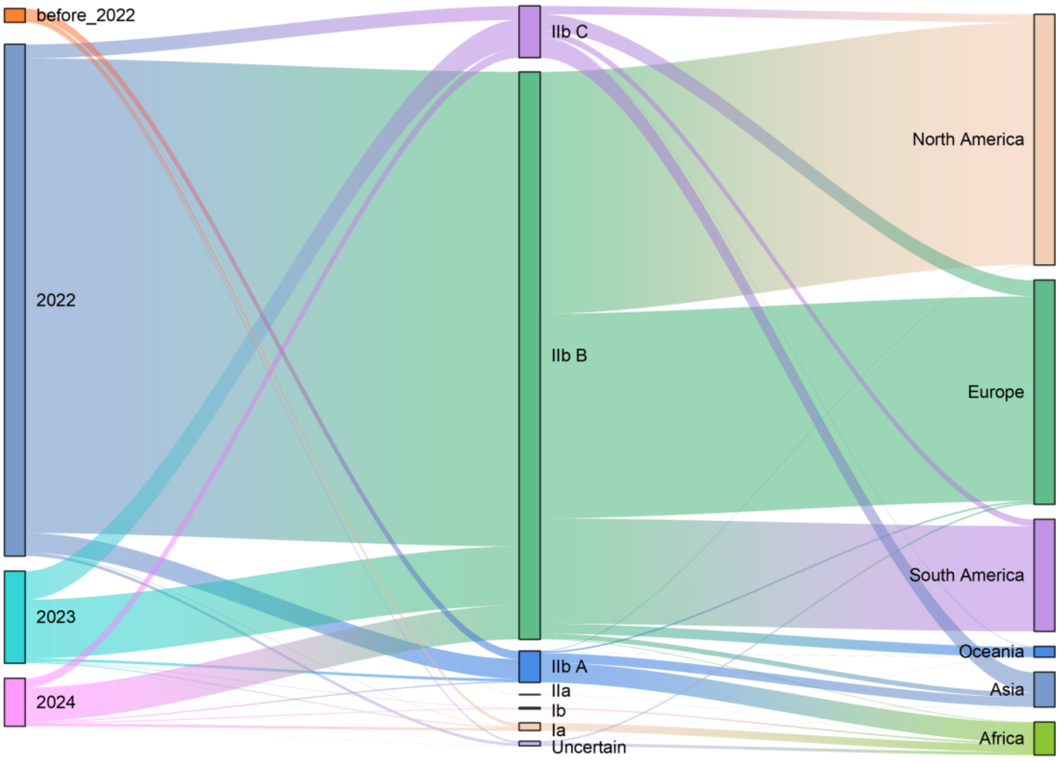

Supplementary Figure 24. Adaptation shift of monkeypox virus samples from clade I and II.

Reduced two components with PCA from the vBERT embedded monkeypox virus samples from clade I and II based on OPG002 **(A)**, OPG015 **(B)**, OPG019 **(C)**, OPG031 **(D)**, OPG034 **(E)**, OPG049 **(F)**, OPG100 **(G)**, OPG130 **(H)**, OPG170 **(I)** and OPG172 **(J)** sequences were visualized respectively. The adaptation shift was also visualized with the arrows.

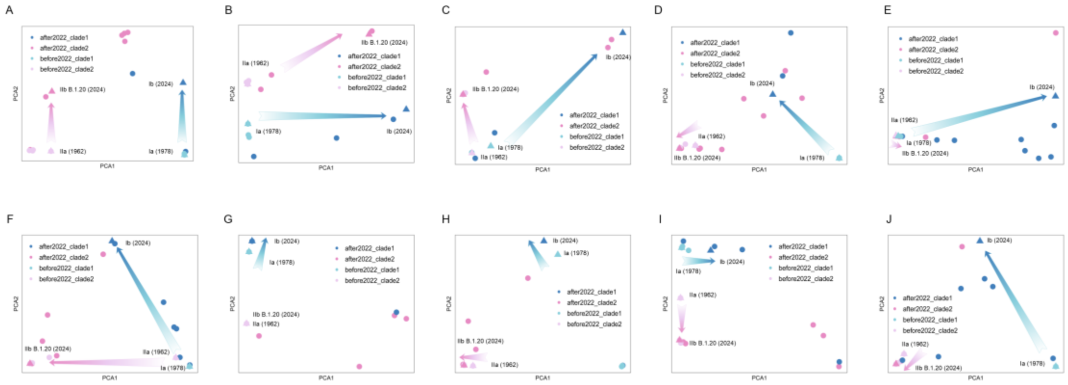

3 Supplementary tables

Supplementary Table 1. Emit probability of HMM.

The emit probability vectors of HMM was optimized for four statuses, respectively Beginning (B), Middle (M), Endding (E) and Single (S).

AA	B	M	E	S
Y	0.041799	0.04026	0.040219	0.01147
W	0.013582	0.013664	0.012694	0.00268
V	0.079302	0.073752	0.077947	0.042721
T	0.071616	0.071619	0.068972	0.03461
S	0.070003	0.07097	0.069995	0.038392
R	0.042368	0.044222	0.044079	0.015569
Q	0.03332	0.035055	0.035167	0.011163
P	0.043708	0.043712	0.044332	0.011584
N	0.049363	0.052365	0.052067	0.018947
M	0.027997	0.023833	0.021821	0.012139
L	0.094739	0.09048	0.09456	0.066398
K	0.057605	0.058548	0.059307	0.021776
I	0.054939	0.054255	0.055752	0.027422
H	0.019902	0.021458	0.021005	0.006736
G	0.06049	0.062328	0.062372	0.020266
F	0.04277	0.045348	0.044208	0.015189
E	0.051259	0.05156	0.051021	0.016679
D	0.053069	0.052391	0.05028	0.017146
C	0.026197	0.02727	0.026493	0.006802
A	0.065971	0.06691	0.067711	0.031313
~	0	0	0	0.570997

Supplementary Table 2. Parameter optimization of language models.

The five clustering indexes (silhouette, CH, DBI, ARI and NMI) of all models involved in parameter optimization were calculated and compared. The optimal indexes were emphasized through bolding.

	Network	vBERT	vBERT	vBERT	vBERT	vBERT	vBERT	vBERT	vBERT	vBERT	vBERT	vBERT
	Parameter	**optimized**	96tokens	no segmentation	whole dataset	simulated dataset	220,000 step	300,000 step	2e-5_lr	2e-3_lr	BERT-tiny	BERT-medium
IAV_HA	silhouette	0.576	0.527	0.527	0.544	0.530	0.556	0.572	0.579	0.499	0.547	**0.585**
	CH	**3348.260**	2747.947	2702.916	2985.517	2726.048	3118.743	3246.409	3056.452	2710.361	2843.703	3307.526
	DBI	**0.580**	0.637	0.640	0.652	0.606	0.648	0.599	0.639	0.681	0.625.	0.607
	ARI	0.834	0.648	0.649	0.763	0.606	0.882	0.775	0.823	0.314	0.773	**0.864**
	NMI	0.905	0.784	0.787	0.857	0.762	0.917	0.872	0.898	0.453	0.869	**0.923**
Spike_RBD	silhouette	0.870	0.859	0.871	**0.878**	0.874	0.866	0.869	0.867	0.860	0.869	0.642
	CH	**14400.057**	13681.561	13327.236	13143.705	13377.535	11093.336	9900.867	12149.422	8991.418	13237.572	1367.386
	DBI	0.215	0.259	0.203	**0.192**	0.207	0.209	0.192	0.216	0.235	0.217	0.497
	ARI	**0.968**	0.953	0.955	0.963	0.946	0.948	0.958	0.955	0.816	0.950	0.850
	NMI	**0.955**	0.936	0.938	0.949	0.926	0.928	0.940	0.938	0.787	0.932	0.855

Supplementary Table 3. High-risk H5N1 mutations predicted by GIVAL with 12 sites mutated.

The mutated amino acids in each of the top 12 sites, mutation sequences and predicted human (HAS) and avian adaptation score (AAS) were listed. The 'Texas_37_2024_H5N1' is the reference strain.

strain_name	strain	HAS	AAS
Texas_37_2024_H5N1	NPANDLCYPGSLNDYEELKHMLSRINHFEKIQIIPKSSWPNHETSLGVSAACPYQGAPSFFRNVVWLIKKNDAYPTIKISYNNTNREDLLILWGIHHSNNAEEQTNLYKNPITYISVGTSTLNQRLAPKIATRSQVNGQRGRMDFFWTILK	0.283105	0.716895
L_93_N|N_100_P|I_118_K|R_149_S|V_151_L|V_152_N|A_160_L|T_192_I|N_193_S|T_200_A|R_212_K|R_227_P	NPANDNCYPGSLPDYEELKHMLSRINHFEKKQIIPKSSWPNHETSLGVSAACPYQGAPSFFSNLNWLIKKNDLYPTIKISYNNTNREDLLILWGIHHSNNAEEQISLYKNPIAYISVGTSTLNQKLAPKIATRSQVNGQPGRMDFFWTILK	0.5001	0.4999
L_93_T|N_100_I|I_118_K|R_149_S|V_151_L|V_152_I|A_160_K|T_192_I|N_193_S|T_200_A|R_212_K|R_227_I	NPANDTCYPGSLIDYEELKHMLSRINHFEKKQIIPKSSWPNHETSLGVSAACPYQGAPSFFSNLIWLIKKNDKYPTIKISYNNTNREDLLILWGIHHSNNAEEQISLYKNPIAYISVGTSTLNQKLAPKIATRSQVNGQIGRMDFFWTILK	0.504467	0.495533
L_93_T|N_100_A|I_118_K|R_149_S|V_151_L|V_152_N|A_160_K|T_192_I|N_193_S|T_200_G|R_212_K|R_227_P	NPANDTCYPGSLADYEELKHMLSRINHFEKKQIIPKSSWPNHETSLGVSAACPYQGAPSFFSNLNWLIKKNDKYPTIKISYNNTNREDLLILWGIHHSNNAEEQISLYKNPIGYISVGTSTLNQKLAPKIATRSQVNGQPGRMDFFWTILK	0.52336	0.47664
L_93_N|N_100_I|I_118_N|R_149_S|V_151_L|V_152_N|A_160_K|T_192_I|N_193_S|T_200_A|R_212_T|R_227_P	NPANDNCYPGSLIDYEELKHMLSRINHFEKNQIIPKSSWPNHETSLGVSAACPYQGAPSFFSNLNWLIKKNDKYPTIKISYNNTNREDLLILWGIHHSNNAEEQISLYKNPIAYISVGTSTLNQTLAPKIATRSQVNGQPGRMDFFWTILK	0.5001	0.4999
L_93_M|N_100_P|I_118_N|R_149	NPANDMCYPGSLPDYEELKHMLSRINHFEKNQIIPKSS	0.501436	0.498564

_S|V_151_L|V_152_N|A_160_K|T	WPNHETSLGVSAACPYQGAPSFFSNLNWLIKKNDKY		
_192_I|N_193_S|T_200_A|R_212	PTIKISYNNTNREDLLILWGIHHSNNAEEQISLYKNPIAY		
_K|R_227_E	ISVGTSTLNQKLAPKIATRSQVNGQEGRMDFFWTILK		
L_93_T|N_100_I|I_118_K|R_149_	NPANDTCYPGSLIDYEELKHMLSRINHFEKKQIIPKSS	0.51433	0.48567
S|V_151_L|V_152_N|A_160_L|T_	WPNHETSLGVSAACPYQGAPSFFSNLNWLIKKNDLYP		
192_I|N_193_S|T_200_A|R_212_	TIKISYNNTNREDLLILWGIHHSNNAEEQISLYKNPIAYI		
K|R_227_P	SVGTSTLNQKLAPKIATRSQVNGQPGRMDFFWTILK		
L_93_T|N_100_P|I_118_N|R_149	NPANDTCYPGSLPDYEELKHMLSRINHFEKNQIIPKSS	0.53119	0.46881
_S|V_151_L|V_152_N|A_160_K|T	WPNHETSLGVSAACPYQGAPSFFSNLNWLIKKNDKY		
_192_I|N_193_S|R_212_T|R_227	PTIKISYNNTNREDLLILWGIHHSNNAEEQISLYKNPITY		
_P	ISVGTSTLNQTLAPKIATRSQVNGQPGRMDFFWTILK		
L_93_T|N_100_P|I_118_N|R_149	NPANDTCYPGSLPDYEELKHMLSRINHFEKNQIIPKSS	0.5001	0.4999
_K|V_151_L|V_152_N|A_160_L|T	WPNHETSLGVSAACPYQGAPSFFKNLNWLIKKNDLYP		
_192_I|N_193_S|R_212_T|R_227	TIKISYNNTNREDLLILWGIHHSNNAEEQISLYKNPITYI		
_P	SVGTSTLNQTLAPKIATRSQVNGQPGRMDFFWTILK		
L_93_T|N_100_P|I_118_N|R_149	NPANDTCYPGSLPDYEELKHMLSRINHFEKNQIIPKSS	0.5001	0.4999
_K|V_151_L|V_152_N|A_160_K|T	WPNHETSLGVSAACPYQGAPSFFKNLNWLIKKNDKY		
_192_I|N_193_S|T_200_G|R_212	PTIKISYNNTNREDLLILWGIHHSNNAEEQISLYKNPIGY		
_T|R_227_P	ISVGTSTLNQTLAPKIATRSQVNGQPGRMDFFWTILK		
L_93_N|N_100_A|I_118_K|R_149	NPANDNCYPGSLADYEELKHMLSRINHFEKKQIIPKSS	0.552456	0.447544
_S|V_151_L|V_152_N|A_160_K|T	WPNHETSLGVSAACPYQGAPSFFSNLNWLIKKNDKY		
_192_I|N_193_S|T_200_A|R_212	PTIKISYNNTNREDLLILWGIHHSNNAEEQISLYKNPIAY		
_T|R_227_P	ISVGTSTLNQTLAPKIATRSQVNGQPGRMDFFWTILK		
L_93_N|N_100_A|I_118_N|R_149	NPANDNCYPGSLADYEELKHMLSRINHFEKNQIIPKSS	0.5001	0.4999
_S|V_151_L|V_152_N|A_160_K|T	WPNHETSLGVSAACPYQGAPSFFSNLNWLIKKNDKY		
_192_I|N_193_S|T_200_A|R_212	PTIKISYNNTNREDLLILWGIHHSNNAEEQISLYKNPIAY		
_K|R_227_P	ISVGTSTLNQKLAPKIATRSQVNGQPGRMDFFWTILK		
L_93_K|N_100_I|I_118_N|R_149_	NPANDKCYPGSLIDYEELKHMLSRINHFEKNQIIPKSS	0.503947	0.496053

S|V_151_L|V_152_N|A_160_L|T_	WPNHETSLGVSAACPYQGAPSFFSNLNWLIKKNDLYP		
192_I|N_193_S|T_200_A|R_212_	TIKISYNNTNREDLLILWGIHHSNNAEEQISLYKNPIAYI		
K|R_227_P	SVGTSTLNQKLAPKIATRSQVNGQPGRMDFFWTILK		
L_93_T|N_100_P|I_118_K|R_149	NPANDTCYPGSLPDYEELKHMLSRINHFEKKQIIPKSS	0.521024	0.478976
_K|V_151_L|V_152_N|A_160_L|T	WPNHETSLGVSAACPYQGAPSFFKNLNWLIKKNDLYP		
_192_I|N_193_S|T_200_A|R_212	TIKISYNNTNREDLLILWGIHHSNNAEEQISLYKNPIAYI		
_T|R_227_P	SVGTSTLNQTLAPKIATRSQVNGQPGRMDFFWTILK		
L_93_T|N_100_P|I_118_F|R_149_	NPANDTCYPGSLPDYEELKHMLSRINHFEKFQIIPKSS	0.5001	0.4999
S|V_151_L|V_152_N|A_160_L|T_	WPNHETSLGVSAACPYQGAPSFFSNLNWLIKKNDLYP		
192_I|N_193_S|T_200_A|R_212_	TIKISYNNTNREDLLILWGIHHSNNAEEQISLYKNPIAYI		
T|R_227_P	SVGTSTLNQTLAPKIATRSQVNGQPGRMDFFWTILK		
L_93_T|N_100_I|I_118_N|V_151_	NPANDTCYPGSLIDYEELKHMLSRINHFEKNQIIPKSS	0.5001	0.4999
L|V_152_N|A_160_K|T_192_I|N_1	WPNHETSLGVSAACPYQGAPSFFRNLNWLIKKNDKY		
93_S|R_212_T|R_227_P	PTIKISYNNTNREDLLILWGIHHSNNAEEQISLYKNPITY		
	ISVGTSTLNQTLAPKIATRSQVNGQPGRMDFFWTILK		
L_93_T|N_100_P|I_118_F|R_149_	NPANDTCYPGSLPDYEELKHMLSRINHFEKFQIIPKSS	0.5001	0.4999
K|V_151_L|V_152_N|A_160_K|T_	WPNHETSLGVSAACPYQGAPSFFKNLNWLIKKNDKY		
192_I|N_193_S|T_200_A|R_212_	PTIKISYNNTNREDLLILWGIHHSNNAEEQISLYKNPIAY		
T|R_227_P	ISVGTSTLNQTLAPKIATRSQVNGQPGRMDFFWTILK		
L_93_T|N_100_P|I_118_K|R_149	NPANDTCYPGSLPDYEELKHMLSRINHFEKKQIIPKSS	0.53518	0.46482
_S|V_151_L|V_152_N|A_160_K|T	WPNHETSLGVSAACPYQGAPSFFSNLNWLIKKNDKY		
_192_I|N_193_S|T_200_A|R_212	PTIKISYNNTNREDLLILWGIHHSNNAEEQISLYKNPIAY		
_T|R_227_P	ISVGTSTLNQTLAPKIATRSQVNGQPGRMDFFWTILK		
L_93_N|N_100_I|I_118_K|R_149_	NPANDNCYPGSLIDYEELKHMLSRINHFEKKQIIPKSS	0.509578	0.490422
S|V_151_L|V_152_N|A_160_L|T_	WPNHETSLGVSAACPYQGAPSFFSNLNWLIKKNDLYP		
192_I|N_193_S|T_200_A|R_212_	TIKISYNNTNREDLLILWGIHHSNNAEEQISLYKNPIAYI		
K|R_227_P	SVGTSTLNQKLAPKIATRSQVNGQPGRMDFFWTILK		
L_93_T|N_100_I|I_118_K|R_149_	NPANDTCYPGSLIDYEELKHMLSRINHFEKKQIIPKSS	0.512233	0.487767

S|V_151_L|V_152_N|A_160_K|T_	WPNHETSLGVSAACPYQGAPSFFSNLNWLIKKNDKY		
192_I|N_193_S|T_200_A|R_212_	PTIKISYNNTNREDLLILWGIHHSNNAEEQISLYKNPIAY		
K|R_227_P	ISVGTSTLNQKLAPKIATRSQVNGQPGRMDFFWTILK		
L_93_N|N_100_M|I_118_F|R_149	NPANDNCYPGSLMDYEELKHMLSRINHFEKFQIIPKSS	0.574424	0.425576
_K|V_151_L|V_152_N|A_160_S|T	WPNHETSLGVSAACPYQGAPSFFKNLNWLIKKNDSY		
_192_I|N_193_S|T_200_G|R_212	PTIKISYNNTNREDLLILWGIHHSNNAEEQISLYKNPIGY		
_K|R_227_E	ISVGTSTLNQKLAPKIATRSQVNGQEGRMDFFWTILK		
L_93_N|N_100_P|I_118_F|R_149	NPANDNCYPGSLPDYEELKHMLSRINHFEKFQIIPKSS	0.5001	0.4999
_S|V_151_L|V_152_N|A_160_K|T	WPNHETSLGVSAACPYQGAPSFFSNLNWLIKKNDKY		
_192_I|N_193_S|T_200_G|R_212	PTIKISYNNTNREDLLILWGIHHSNNAEEQISLYKNPIGY		
_T|R_227_P	ISVGTSTLNQTLAPKIATRSQVNGQPGRMDFFWTILK		
L_93_S|N_100_P|I_118_F|R_149	NPANDSCYPGSLPDYEELKHMLSRINHFEKFQIIPKSS	0.5001	0.4999
_K|V_151_L|V_152_I|A_160_K|T_	WPNHETSLGVSAACPYQGAPSFFKNLIWLIKKNDKYP		
192_I|N_193_S|T_200_A|R_212_	TIKISYNNTNREDLLILWGIHHSNNAEEQISLYKNPIAYI		
T|R_227_P	SVGTSTLNQTLAPKIATRSQVNGQPGRMDFFWTILK		
L_93_I|N_100_P|I_118_K|R_149_	NPANDICYPGSLPDYEELKHMLSRINHFEKKQIIPKSS	0.522478	0.477522
S|V_151_L|V_152_N|A_160_K|T_	WPNHETSLGVSAACPYQGAPSFFSNLNWLIKKNDKY		
192_I|N_193_S|T_200_A|R_212_	PTIKISYNNTNREDLLILWGIHHSNNAEEQISLYKNPIAY		
K|R_227_P	ISVGTSTLNQKLAPKIATRSQVNGQPGRMDFFWTILK		
L_93_N|N_100_I|I_118_K|R_149_	NPANDNCYPGSLIDYEELKHMLSRINHFEKKQIIPKSS	0.5001	0.4999
S|V_151_L|V_152_N|A_160_K|T_	WPNHETSLGVSAACPYQGAPSFFSNLNWLIKKNDKY		
192_I|N_193_S|T_200_A|R_212_	PTIKISYNNTNREDLLILWGIHHSNNAEEQISLYKNPIAY		
K|R_227_P	ISVGTSTLNQKLAPKIATRSQVNGQPGRMDFFWTILK		
L_93_T|N_100_M|I_118_N|R_149	NPANDTCYPGSLMDYEELKHMLSRINHFEKNQIIPKSS	0.554184	0.445816
_K|V_151_L|V_152_N|A_160_S|T	WPNHETSLGVSAACPYQGAPSFFKNLNWLIKKNDSY		
_192_I|N_193_S|T_200_A|R_212	PTIKISYNNTNREDLLILWGIHHSNNAEEQISLYKNPIAY		
_K|R_227_E	ISVGTSTLNQKLAPKIATRSQVNGQEGRMDFFWTILK		
L_93_N|N_100_A|I_118_K|R_149	NPANDNCYPGSLADYEELKHMLSRINHFEKKQIIPKSS	0.5001	0.4999

_K|V_151_L|V_152_N|A_160_K|T	WPNHETSLGVSAACPYQGAPSFFKNLNWLIKKNDKY		
_192_I|N_193_S|T_200_G|R_212	PTIKISYNNTNREDLLILWGIHHSNNAEEQISLYKNPIGY		
_T|R_227_P	ISVGTSTLNQTLAPKIATRSQVNGQPGRMDFFWTILK		
L_93_T|N_100_P|I_118_N|R_149	NPANDTCYPGSLPDYEELKHMLSRINHFEKNQIIPKSS	0.5001	0.4999
_S|V_151_L|V_152_N|A_160_K|T	WPNHETSLGVSAACPYQGAPSFFSNLNWLIKKNDKY		
_192_I|N_193_S|T_200_G|R_212	PTIKISYNNTNREDLLILWGIHHSNNAEEQISLYKNPIGY		
_T|R_227_P	ISVGTSTLNQTLAPKIATRSQVNGQPGRMDFFWTILK		
L_93_T|N_100_I|I_118_K|R_149_	NPANDTCYPGSLIDYEELKHMLSRINHFEKKQIIPKSS	0.5001	0.4999
S|V_151_L|V_152_I|A_160_K|T_1	WPNHETSLGVSAACPYQGAPSFFSNLIWLIKKNDKYP		
92_I|N_193_S|T_200_A|R_212_T|	TIKISYNNTNREDLLILWGIHHSNNAEEQISLYKNPIAYI		
R_227_P	SVGTSTLNQTLAPKIATRSQVNGQPGRMDFFWTILK		
L_93_T|N_100_P|I_118_K|R_149	NPANDTCYPGSLPDYEELKHMLSRINHFEKKQIIPKSS	0.5001	0.4999
_S|V_151_L|V_152_N|A_160_T|T	WPNHETSLGVSAACPYQGAPSFFSNLNWLIKKNDTYP		
_192_I|N_193_S|T_200_A|R_212	TIKISYNNTNREDLLILWGIHHSNNAEEQISLYKNPIAYI		
_T|R_227_P	SVGTSTLNQTLAPKIATRSQVNGQPGRMDFFWTILK		
L_93_T|N_100_P|I_118_K|R_149	NPANDTCYPGSLPDYEELKHMLSRINHFEKKQIIPKSS	0.5001	0.4999
_S|V_151_L|V_152_N|A_160_L|T	WPNHETSLGVSAACPYQGAPSFFSNLNWLIKKNDLYP		
_192_I|N_193_S|T_200_A|R_212	TIKISYNNTNREDLLILWGIHHSNNAEEQISLYKNPIAYI		
_K|R_227_P	SVGTSTLNQKLAPKIATRSQVNGQPGRMDFFWTILK		
L_93_N|N_100_P|I_118_N|R_149	NPANDNCYPGSLPDYEELKHMLSRINHFEKNQIIPKSS	0.5001	0.4999
_S|V_151_L|V_152_N|A_160_K|T	WPNHETSLGVSAACPYQGAPSFFSNLNWLIKKNDKY		
_192_I|N_193_S|T_200_A|R_212	PTIKISYNNTNREDLLILWGIHHSNNAEEQISLYKNPIAY		
_T|R_227_P	ISVGTSTLNQTLAPKIATRSQVNGQPGRMDFFWTILK		
L_93_T|N_100_P|I_118_F|R_149_	NPANDTCYPGSLPDYEELKHMLSRINHFEKFQIIPKSS	0.505424	0.494576
S|V_151_L|V_152_N|A_160_K|T_	WPNHETSLGVSAACPYQGAPSFFSNLNWLIKKNDKY		
192_I|N_193_D|R_212_T|R_227_	PTIKISYNNTNREDLLILWGIHHSNNAEEQIDLYKNPITY		
P	ISVGTSTLNQTLAPKIATRSQVNGQPGRMDFFWTILK		
L_93_N|N_100_P|I_118_F|R_149	NPANDNCYPGSLPDYEELKHMLSRINHFEKFQIIPKSS	0.5001	0.4999

_S|V_151_L|V_152_N|A_160_K|T<br>_192_I|N_193_S|T_200_A|R_212<br>_T|R_227_P	WPNHETSLGVSAACPYQGAPSFFSNLNWLIKKNDKY<br>PTIKISYNNTNREDLLILWGIHHSNNAEEQISLYKNPIAY<br>ISVGTSTLNQTLAPKIATRSQVNGQPGRMDFFWTILK		
L_93_T|N_100_P|I_118_K|R_149<br>_S|V_151_L|V_152_N|A_160_L|T<br>_192_I|N_193_S|T_200_G|R_212<br>_T|R_227_P	NPANDTCYPGSLPDYEELKHMLSRINHFEKKQIIPKSS<br>WPNHETSLGVSAACPYQGAPSFFSNLNWLIKKNDLYP<br>TIKISYNNTNREDLLILWGIHHSNNAEEQISLYKNPIGYI<br>SVGTSTLNQTLAPKIATRSQVNGQPGRMDFFWTILK	0.519393	0.480607

Supplementary Table 4. High-risk H5N1 mutations predicted by GIVAL with 8 sites mutated.

The mutated amino acids in each of the top 8 sites, mutation sequences and predicted human (HAS) and avian adaptation score (AAS) were listed. The 'Texas_37_2024_H5N1' is the reference strain.

strain_name	strain	HAS	AAS
Texas_37_2024_H5N1	NPANDLCYPGSLNDYEELKHMLSRINHFEKIQIIPKSS<br>WPNHETSLGVSAACPYQGAPSFFRNVVWLIKKNDAY<br>PTIKISYNNTNREDLLILWGIHHSNNAEEQTNLYKNPIT<br>YISVGTSTLNQRLAPKIATRSQVNGQRGRMDFFWTILK	0.283105	0.716895
I_118_K|V_151_L|V_152_<br>N|A_160_T|T_192_I|N_19<br>3_S|R_212_K|R_227_F	NPANDLCYPGSLNDYEELKHMLSRINHFEKKQIIPKSS<br>WPNHETSLGVSAACPYQGAPSFFRNLNWLIKKNDTY<br>PTIKISYNNTNREDLLILWGIHHSNNAEEQISLYKNPITY<br>ISVGTSTLNQKLAPKIATRSQVNGQFGRMDFFWTILK	0.5001	0.4999
I_118_F|V_151_L|V_152_<br>I|A_160_L|T_192_I|N_193<br>_S|R_212_K|R_227_F	NPANDLCYPGSLNDYEELKHMLSRINHFEKFQIIPKSS<br>WPNHETSLGVSAACPYQGAPSFFRNLIWLIKKNDLYP<br>TIKISYNNTNREDLLILWGIHHSNNAEEQISLYKNPITYI<br>SVGTSTLNQKLAPKIATRSQVNGQFGRMDFFWTILK	0.522527	0.477473

Supplementary Table 5. Circulating IAV H5N1 HA RBD sequences corresponding to the high-risk mutations.

The strain name, HA RBD sequences and high-risk mutations of circulating IAV H5N1 collected in 2025.

<table>
<tr><th>seq_id</th><th>seq</th><th>site_ mutation</th></tr>
<tr><td>A/dairy_cow/USA/003670-005/2025|EPI_ISL_19743017|A_/_H5N1|Original||2.3.4.4b|2025-01-01|HA|EPI4000598</td><td>NPANGLCYPGSLNDYEELKHMLSRINHFEKIQIIPKSSWPNHETSLGVSAACPYQGAPSFFRNVVWLIKKNDSYPTIKKSYNNTNREDLLILWGIHHSNNAEEQTNLYKNPITYISVGTSTLNQRLAPKIATRSQVNGQRGRMDFFWTILK</td><td>160_S</td></tr>
<tr><td>A/dairy_cow/California/038628-001/2024|EPI_ISL_19661068|A_/_H5N1|Original||2.3.4.4b|2024-12-23|HA|EPI3785124</td><td>NPANGLCYPGSLNDYEELKHMLSRINHFEKIQIIPKSSWPNHETSLGVSAACPYQGAPSFFRNVVWLIKKNDAYPTIKISYNNTNREDLLILWGIHHSNNAEEQTNLYKNPITYISVGTSTLNQKLAPRIATRSQVNGQRGRMDFFWTILK</td><td>212_K</td></tr>
<tr><td>A/dairy_cow/USA/007915-001/2025|EPI_ISL_19792255|A_/_H5N1|Original||2.3.4.4b|2025-01-01|HA|EPI4148573</td><td>NPANGLCYPGSLNDYEELKHMLSRINHFEKIQIIPKSSWPNHETSLGVSAACPYQGAPSFFRNVVWLIKKNDAYPTIKISYNNTNREDLLILWGIHHSNNAKEQTNLYKNPITYISVGTSTLNQKLAPRIATRSQVNGQRGRMDFFWTILK</td><td>212_K</td></tr>
<tr><td>A/dairy_cow/California/038334-002/2024|EPI_ISL_19661121|A_/_H5N1|Original||2.3.4.4b|2024-12-17|HA|EPI3785532</td><td>NPANGLCYPGSLNDYEELKHMLSRINHFEKIQIIPKSSWPNHETSLGVSAACPYQGAPSFFRNVVWLIKKNDTYPTIKISYNNTNREDLLILWGIHHSNNAEEQTNLYKNPTTYISVGTSTLNQRLAPKIATRSQVNGQRGRMDFFWTILK</td><td>160_T</td></tr>
<tr><td>A/vulture/USA/006842-001/2025|EPI_ISL_19792303|A_/_H5N1|Original||2.3.4.4b|2025-01-01|HA|EPI4148931</td><td>NPANDLCYPGSLNDYEELKHLLSRINHFEKILIIPKSSWPNHETSLGVSAACPYQGAPSFFRNVVWLIKKNDAYPTIKISYNNTNREDLLILWGIHHSNNAEEQTNLYKNPTAYISVGTSTLNQRLVPKIATRSQVNGQRGRMDFFWTILK</td><td>200_A</td></tr>
<tr><td>A/turkey/Iowa/25-005292-002-R2/2025|EPI_ISL_19873850|A_/_H5N1|Original||2.3.4.4b|2025-02-12|HA|EPI4367914</td><td>NPANDLCYPGSLNDYEELKHLLSRINHFEKILIIPKSSWPNHETSLGVSAACPYQGAPSFFRNLVWLIKKNDAYPTIKISYNNTNREDLLILWGIHHSNNAEEQTNLYKNPTTYISVGTSTLNQRLVPKIATRSQVNGQRGRMDFFWTILK</td><td>151_L</td></tr>
<tr><td>A/chicken/Nebraska/25-005224-001-R2/2025|EPI_ISL_19873881|A_/_H5N1|Original||2.3.4.4b|2025-02-11|HA|EPI4368162</td><td>NPANDLCYPGSLNDYEELKHLLSRINHFEKILIIPKSSWPNHETSLGVSAACPYQGAPSFFRNVVWLIKKNDAYPTIKISYNNTNREDLLILWGIHHSNNAEEQINLYKNPTTYISVGTSTLNQRLVPKIATRSQVNGQRGRMDFFWTILK</td><td>192_I</td></tr>
<tr><td>A/Cambodia/KSH250004/2025|EPI_ISL_19661054|A_/_H5N1|Original||2.3.2.1e|2025-01-08|HA|EPI3785019</td><td>NPANDLCYPGNFNDYEELKHLLSRINHFEKIQIIPKNSWSDHEASLGVSAACSYQGNSSFFRNVVWLIKKDNTYPTIKKDYNNTNREDLLILWGIHHPNDEAEQTKLYQNPTTYISIGTSTLNQRLVPKIATRPKINGQSGRIDFFWTILK</td><td>160_T</td></tr>
</table>

A/european_herring_gull/Italy/24VIR9146-2/2024|EPI_ISL_19529924|A_/_H5N1|||2.3.4.4b|2024-10-08|HA|EPI3627963	NPANDLCYPGSLNDYEELKHLLSRINHFEKILIIPKSSWPNHETSLGVSAACSYQGAPSFFRNVVWLIKKNDAYPTIKISYNNTNREDLLILWGIHHSNNAEEQTSLYKNPATYISVGTSTLNQRLVPKIATRSQVNGQRGRMDFFWTILK	193_S
A/dairy_cow/USA/010490-001/2025|EPI_ISL_19825637|A_/_H5N1|Original||2.3.4.4b|2025-01-01|HA|EPI4243853	NPANGLCYPGSLNDYEELKHMLSRINHFEKIQIIPKSSWPNHETSLGVSAACPYQGAPSFFRNVVWLIKKNDAYPTIKISYNNTNREDLLILWGIHHSNNAKEQTNLYKNPITYISVGTSTLNQKLTPRIATRSQVNGQRGRMDFFWTILK	212_K
A/dairy_cow/USA/010514-002/2025|EPI_ISL_19825634|A_/_H5N1|Original||2.3.4.4b|2025-03-24|HA|EPI4243832	NPANDLCYPGSLNDYEELKHMLSRINHFEKIQIIPKSSWPNHETSLGVSAACPYQGAPSFFRNVVWLIKKNDAYPTIKISYNNTNREDLLILWGIHHSNNAEEQINLYKNPITYISVGTSTLNQRLAPKIATRSQVNGQRGRMDFFWTILK	192_I
A/turkey_vulture/USA/010287-001/2025|EPI_ISL_19825662|A_/_H5N1|Original||2.3.4.4b|2025-01-01|HA|EPI4244035	NPANDLCYPGSLNDYEELKHLLSRINHFEKILIIPKSSWPNHETSLGVSAACPYQGAPSFFRNVVWLIKKNDAYPTIKISYNNTNREDLLILWGIHHSNNAEEQTNLYKNPTTYISVGTSTLNQKLVPKIATRSQVNGQRGRMDFFWTILK	212_K
A/chicken/Czech_Republic/19556-2/2024|EPI_ISL_19661420|A_/_H5N1|original||2.3.4.4b|2024-12-16|HA|EPI3786082	NPANDLCYPGSLNDYEELKHLLSRINHFEKILIIPKSSWPNHETSLGVSAACSYQGAPSFFRNVVWLIKKNDAYPTIKISYNNTNREDLLILWGIHHSNNAEEQINLYKNPATYISVGTSTLNQRLVPKIATRSQVNGQRGRMDFFWTILK	192_I
A/red-tailed_hawk/USA/007119-016/2025|EPI_ISL_19808900|A_/_H5N1|Original||2.3.4.4b|2025-01-01|HA|EPI4196674	NPANDLCYPGSLNDYEELKHLLSRINHFEKILIIPKSSWPNHETSLGVSAACPYQGAPSFFRNVVWLIKKNDTYPTIKISYNNTNREDLLILWGIHHSNNAEEQTNLYKNPTTYISVGTSTLNQRLVPKIATRSQVNGQRGRMDFFWTILK	160_T
A/duck/Burkina_Faso/BKF11_24VIR3019-11/2024|EPI_ISL_19171212|A_/_H5N1|||2.3.4.4b|2024-03-04|HA|EPI3343250	NPANDLCYPGSLNDYEELKHLLSRINHFEKILIIPKSSWPNHETSLGVSAACPYQGAPSFFRNVVWLIKKNDSYPTIKISYNNTNQEDLLILWGIHHSNNAEEQTNLYKNPTTYISVGTSTLNQRLVPKIATRSLVNGQRGRMDFFWTILK	160_S
A/turkey/Burkina_Faso/BKF13_24VIR3019-13/2024|EPI_ISL_19171213|A_/_H5N1|||2.3.4.4b|2024-03-25|HA|EPI3343258	NPANDLCYPGSLNDYEELKHLLSRINHFEKILIIPKSSWPNHETSRGVSAACPYQGAPSFFRNVVWLIKKNDSYPTIKISYNNTNQEDLLILWGIHHSNNAEEQTNLYKNPTTYISVGTSTLNQRLVPKIATRSLVNGQRGRMDFFWTILK	160_S
A/dairy_cow/USA/011973-001/2025|EPI_ISL_19859565|A_/_H5N1|Original||2.3.4.4b|2025-04-03|HA|EPI4314786	NPANDLCYPGSLNDYEELKHMLSKINHFEKIQIIPKSSWPNHEASLGVSAACPYLGAPSFFRNVVWLIKKNNAYPTIKISYNNTNREDLLILWGIHHSNNAEEQINLYKNPITYISVGTSTLNQRLAPKIATRSQVNGQRGRMDFFWTILK	192_I
A/dairy_cow/USA/020928-002-tile/2024|EPI_ISL_19876997|A_/	NPANDLCYPGSLNDYEELKHMLSRINHFEKIQIIPKSSWPNHETSLGVSAACPYQGAPSFFRNVVWLIKKNDSYPTIKISYNNTNREDLLILWGIHH	160_S

_H5N1|Original||2.3.4.4b|2024-01-01|HA|EPI4376178	SNNAEEQTNLYKNPITYISVGTSTLNQRLAPKIATRSQVNGQRGRMDFFWTILK	
A/Victoria/149/2024|EPI_ISL_19156871|A_/_H5N1|SIAT2||2.3.2.1a|2024-03-06|HA|EPI3317786	NPANGLCYPGNFNDYEELKHLLSRINHFEKIRIIPKDSWSDHDASLGVSAACPYQGNPSFFRNVVWLIKKNNSYPTIKKSYNNTNQEDLLILWGIHHPNDEAEQTNLYQNPITYISIGTSTLNQRLIPRIATRSKINGQSGRIDFFWTILK	160_S
A/environment/Kagoshima/KU-24G1/2024(H5N1)|EPI_ISL_19534185|A_/_H5N1|||2.3.4.4b|2024-11-04|HA|EPI3632533	NPANDLCYPGSLNDYEELKHLLSRINHFEKILIIPKSSWSNHETSLGVSAACSYQGAPSFFRNVVWLIKKDDAYPTIKISYNNTNREDLLILWGIHHSNNAEEQTDLYKNPTTYISVGTSTLNQRLVPKIATRSQVNGQRGRMDFFWTILK	193_D
A/chicken/Bangladesh/CDILIV_H5N1_58/2025|EPI_ISL_19894604|A_/_H5N1|Original||2.3.2.1a|2025-03-01|HA|EPI4410422	NPANGLCYPGNFNDYEELKHLLSRINHFEKIRIIPKDSRSDHDASLGVSAACPYHGNPSFFRNVVWLIKKNNSYPTIKESYDHTNQEDLLILWGIHHPNDEAEQTNLYQNPITYISIGTSTLNQRLIPRIATRSKINGQSGRIDFFWTILK	160_S
A/dairy_cow/California/25_000528-001/2024|EPI_ISL_19829275|A_/_H5N1|Original||2.3.4.4b|2024-12-30|HA|EPI4253659	NPANGLCYPGSLNDYEELKHMLSRINHFEKIQIIPKSSWPNHETSLGMSAACPYQGAPSFFRNVVWLIKKNDTYPTIKISYNNTNREDLLILWGIHHSNNAEEQTNLYKNPITYISVGTSTLNQRLAPKIATRSQVNGQRGRMDFFWTILK	160_T
A/Greylag_Goose/England/148684/2025|EPI_ISL_19896379|A_/_H5N1|Clinical_Sample||2.3.4.4b|2025-03-18|HA|EPI4413279	NPANDLCYPGSLNDYEELKHLLSRINHFEKILIIPKSSWPNHETSLGVSAACSYQGAPSFFRNVVWLIKKNDAYPTIKISYNNTNREDLLILWGIHHSNNAEEQTNLYKNPATYISVGTSTLNQKLVPKIATRSQVNGQRGRMDFFWTILK	212_K
A/dairy_cow/Texas/24_009499-001/2024|EPI_ISL_19094455|A_/_H5N1|Original||2.3.4.4b|2024-03-17|HA|EPI3259825	NPANDLCYPGSLNDYEELKHMLSRINHFEKIQIIPKSSWPNHETSLGVSAACPYQGAPSFFRNVVWLIKKNDTYPTIKISYNNTNREDLLILWGIHHSNNAEEQTNLYKNPITYISVGTSTLNQRLAPKIATRSQVNGQRGRMDFFWTILK	160_T
A/domestic-duck/Bulgaria/860_24VIR11025-10/2024|EPI_ISL_19701031|A_/_H5N1|||2.3.4.4b|2024-05-09|HA|EPI3873289	NPANDLCYPGSLNDYEELKHLLSRINHFEKILIIPKSSWSNHETSLGVSAACPYQGAPSFFRNVVWLVKKNNAYPTIKISYNNTNREDLLILWGIHHSNNAKEQTDLYKNPTTYISVGTSTLNQRLVPKITTRSQVNGQRGRMDFFWTILK	193_D
A/dairy_cow/USA/006026-001/2025|EPI_ISL_19832237|A_/_H5N1|Original||2.3.4.4b|2025-01-01|HA|EPI4255898	NPANGLCYPGSLNDYEELKHMLSRINHFEKIQIIPKSSWPNHETSLGVSAACPYQGAPSFFRNVVWLIKKNDTYPTIKISYNNTNREDLLILWGIHHSNNAEEQTNLYKNPITYISVGTSTLNQRLAPKIATRSQVNGQRGRMDFFWTILK	160_T
A/dairy_cow/USA/028767-001-tile/2024|EPI_ISL_19882317|A_/_H5N1|Original||2.3.4.4b|2024-01-01|HA|EPI4388719	NPANGLCYPGSLNDYEELKHMLSRINHFEKIQIIPKSSWPNHETSLGVSAACPYQGAPSFFRNVVWLIKKNDAYPTIKISYNNTNREDLLILWGIHHSNNAEEQTDLYKNPITYISVGTSTLNQRLAPKIATRSQVNGQRGRMDFFWTILK	193_D
A/dairy_cow/Texas/24_015837-002-	NPANDLCYPGSLNDYEELKHMLSRINHFEKIQIIPKSSWPNHETSLGVSAACSYQGAPSFFRNVVWLIKKNDTYPTIKISYNNTNREDLLILWGIHH	160_T

R2/2024|EPI_ISL_19310339|A_/_H5N1|Original||2.3.4.4b|2024-04-08|HA|EPI3488167	SNNAEEQTNLYKNPITYISVGTSTLNQRLAPKIATRSQVNGQRGRMDFFWTILK	
A/turkey_vulture/Massachusetts/014126-068/2024|EPI_ISL_19736871|A_/_H5N1|Original||2.3.4.4b|2024-05-03|HA|EPI3980567	NPANDLCYPGSLNDYEELKHLLSRINHFEKILIIPKGSWPNHDTSLGVSAACPYQGAPSFFRNVVWLIKKNDTYPTIKISYNNTNREDLLILWGIHHSNNAEEQTNLYKNPTTYISVGTSTLNQRLVPKIATRSQVNGQRGRMDFFWTILK	160_T
A/mallard/Italy/24VIR9197-2/2024|EPI_ISL_19540011|A_/_H5N1|||2.3.4.4b|2024-10-07|HA|EPI3637556	NPANDLCYPGSLNDYEELKHLLSRINHFEKILIIPKSSWPNHETSLGVSAACSYQGAPSFFRNVVWLIKKNDAYPTIKISYNNTNREDLLILWGIHHSNNAKEQTSLYKNPATYISVGTSTLNQRLVPKIATRSQVNGQRGRMDFFWTILK	193_S
A/dairy_cow/USA/015677-001/2025|EPI_ISL_19900960|A_/_H5N1|Original||2.3.4.4b|2025-01-01|HA|EPI4428495	NPANGLCYPGSLNDYEELKHMLSRINHFEKIQIIPKSSWPNHETSLGVSAACPYQGAPSFFRNVVWLIKKNDTYPTIKISYNNTNREDLLILWGIHHSNNAEEQTNLYKNPITYISVGTSTLNQKLAPRIATRSQVNGQRGRMDFFWTILK	160_T|212_K
A/Larus_michaellis/Spain/3268-15_24VIR10678-33/2024|EPI_ISL_19624113|A_/_H5N1|||2.3.4.4b|2024-09-18|HA|EPI3729815	NPANGLCYPGSLNDYEELKHLLSRINHFEKILIIPKSSWPNHETSLGVSAACPYQGAPSFFRNVVWLIKKNDAYPTIKISYNNTNREDLLILWGIHHSNNAEEQINLYKNPTTYISVGTSTLNQRLVPKIATRSQVNGQRGRMDFFWTILK	192_I
